# Association between cigarette smoking status, intensity, and cessation duration with long-term incidence of nine cardiovascular and mortality outcomes: The Cross-Cohort Collaboration (CCC)

**DOI:** 10.1371/journal.pmed.1004561

**Published:** 2025-11-18

**Authors:** Erfan Tasdighi, Zhiqi Yao, Zeina A. Dardari, Kunal K. Jha, Ngozi Osuji, Tanuja Rajan, Ellen Boakye, Kunihiro Matsushita, Eleanor M. Simonsick, Joao A. C. Lima, Donald M. Lloyd-Jones, Debbie L. Cohen, Lawrence J. Appel, Amit Khera, Michael E. Hall, Carlos J. Rodriguez, Suzanne Judd, Shelley A. Cole, Vasan S. Ramachandran, Emelia J. Benjamin, Paulo A. Lotufo, Marcio Sommer Bittencourt, Samar R. El Khoudary, Rebecca C. Thurston, Carol A. Derby, Bruce M. Psaty, Charles B. Eaton, Michael J. LaMonte, Peggy M. Cawthon, Eric S. Orwoll, Aruni Bhatnagar, Andrew P. DeFilippis, Michael J. Blaha

**Affiliations:** 1 Johns Hopkins Ciccarone Center for Prevention of Cardiovascular Disease, Baltimore, Maryland, United States of America; 2 Department of Internal Medicine, NJMS Medical School, Rutgers Health University, Newark, New Jersey, United States of America; 3 University of Louisville School of Medicine, Louisville, Kentucky, United States of America; 4 Department of Medicine, University of Pittsburgh Medical Center, Pittsburgh, Pennsylvania, United States of America; 5 Department of Medicine, Perelman School of Medicine, University of Pennsylvania, Philadelphia, Pennsylvania, United States of America; 6 The American Heart Association Tobacco Regulation and Addiction Center, Dallas, Texas, United States of America; 7 Department of Epidemiology, Welch Center for Prevention, Epidemiology, and Clinical Research, Johns Hopkins Bloomberg School of Public Health, Baltimore, Maryland, United States of America; 8 Intramural Research Program, National Institute on Aging, Baltimore, Maryland, United States of America; 9 Division of Cardiology, Department of Medicine, Johns Hopkins University, Baltimore, Maryland, United States of America; 10 Division of Cardiology, Department of Medicine, Northwestern University Feinberg School of Medicine, Chicago, Illinois, United States of America; 11 Renal, Electrolyte and Hypertension Division, Perelman School of Medicine, University of Pennsylvania, Philadelphia, Pennsylvania, United States of America; 12 University of Texas Southwestern, Division of Cardiology, Dallas, Texas, United States of America; 13 Department of Medicine, University of Mississippi Medical Center, Jackson, Mississippi, United States of America; 14 Department of Cardiology, Albert Einstein College of Medicine, New York, New York, United States of America; 15 Department of Biostatistics, University of Alabama at Birmingham School of Public Health, Birmingham, Alabama, United States of America; 16 Population Health Program, Texas Biomedical Research Institute, San Antonio, Texas, United States of America; 17 University of Texas School of Public Health, San Antonio, Texas, United States of America; 18 Department of Epidemiology, Boston University School of Public Health, Boston, Massachusetts, United States of America; 19 Universidade de São Paulo, Center for Clinical and Epidemiological Research, São Paulo, Brazil; 20 Department of Internal Medicine, Universidade de São Paulo, São Paulo, Brazil; 21 Department of Cardiology, University of Pittsburgh Medical Center, Pittsburgh, Pennsylvania, United States of America; 22 Department of Epidemiology, Graduate School of Public Health, University of Pittsburgh, Pittsburgh, Pennsylvania, United States of America; 23 Department of Psychiatry, University of Pittsburgh School of Medicine, Pittsburgh, Pennsylvania, United States of America; 24 Department of Neurology, Albert Einstein College of Medicine, New York, New York, United States of America; 25 Cardiovascular Health Research Unit, Departments of Medicine, Epidemiology, and Health Systems and Population Health, University of Washington, Seattle, Washington, United States of America; 26 Center for Primary Care and Prevention, Department of Family Medicine, Department of Epidemiology, Warren Alpert Medical School of Brown University, Providence, Rhode Island, United States of America; 27 Department of Epidemiology, Brown University School of Public Health, Providence, Rhode Island, United States of America; 28 Department of Epidemiology and Environmental Health, School of Public Health and Health Professions, University at Buffalo, Buffalo, New York, United States of America; 29 California Pacific Medical Center Research Institute, San Francisco, California, United States of America; 30 Division of Endocrinology, Diabetes and Clinical Nutrition, School of Medicine, Oregon Health & Science University, Portland, Oregon, United States of America; 31 Department of Medicine, Vanderbilt University Medical Center, Nashville, Tennessee, United States of America; Australian National University, AUSTRALIA

## Abstract

**Background:**

Uncertainties persist regarding the precise shape of the smoking-outcome curves across various cardiovascular and mortality endpoints. This study aims to elucidate the relationships among smoking burden, intensity, and cessation duration across multiple cardiovascular outcomes.

**Methods and findings:**

Cox proportional hazard models were constructed to evaluate the association between pack-years, cigarettes per day (CPD), and years since cessation with cardiovascular outcomes in participants from 22 prospective cohort studies within the Cross-Cohort Collaboration Tobacco Working Group. We evaluated myocardial infarction (MI), stroke, coronary heart disease (CHD; MI, coronary revascularization, or coronary death), cardiovascular disease (CVD; stroke or cardiovascular death), heart failure (HF), atrial fibrillation (AFib), CHD mortality, CVD mortality, and all-cause mortality. Median follow-up varied across outcomes, with 14.4 years for MI (17,570 events), 19.3 years for CHD (30,625 events), 18.6 years for CVD (54,078 events), and approximately 19.4–19.9 years for mortality outcomes (CHD mortality: 17,429 events; CVD mortality: 33,120 events; all-cause mortality: 125,044 events). Spline terms were used to investigate the nonlinear association of continuous smoking/cessation measures with the examined outcomes. Models were adjusted for demographic, socioeconomic, and other cardiovascular risk factors. The study included 323,826 adults (148,635 non-mortality and 176,396 mortality outcomes with 25 and 16 million person-years at risk, respectively). Compared to never-smokers, current smokers had significantly increased risks for CVD (hazard ratio (HR) 1.74, 95% confidence intervals (CIs) [1.66,1.83] in men; HR 2.07, 95% CI [2.00,2.14] in women) and all-cause mortality (HR 2.17, 95% CI [2.09,2.25] in men; HR 2.43, 95% CI [2.38,2.48] in women; all *p* < 0.001). Compared with never-smokers, participants with 2–5 CPD demonstrated substantially elevated cardiovascular risks, with HR ranging from 1.26 (95% CI [1.09,1.45], *p* = 0.002) for AFib to 1.57 (95% CI [1.39,1.78], *p* < 0.001) for HF. Smoking 2–5 CPD was associated with increased CVD mortality (HR 1.57, 95% CI [1.41,1.75]), and all-cause mortality (HR 1.60, 95% CI [1.52,1.69]; both *p* < 0.001). Smoking 11–15 CPD conferred a higher risk of CVD (HR 1.87, 95% CI [1.69,2.06]) and all-cause mortality (HR 2.30, 95% CI [2.14,2.47]; both *p* < 0.001). The increased risk associated with the evaluated outcomes was steeper for the initial 20 pack-years and 20 CPD, respectively, compared to further smoking exposure. The most substantial reduction in risk across all outcomes was observed within the first 10 years after smoking cessation. However, the progressive risk reduction continues over extended time periods, with former smokers demonstrating over 80% lower relative risk than those of current smokers within 20 years of cessation. Limitations include potential exposure misclassification due to reliance on single baseline self-reported smoking measurements with extended follow-up periods, which may underestimate true risk associations, and lack of data on other tobacco products and electronic nicotine delivery systems, preventing analysis of dual- and poly-use patterns.

**Conclusion:**

Lower-intensity smoking is associated with cardiovascular risk and the primary public health message for current smokers should be early cessation, rather than reducing the amount of smoking. Cessation provides substantial immediate risk reduction, although risk continues to decrease significantly for the following two decades.

## Author summary

**Why was this study done?:**

- Previous research established that cigarette smoking increases cardiovascular disease risk, but the exact relationship between smoking intensity and health outcomes remained unclear, especially for low-intensity smoking.
- Studies reported inconsistent timeframes—ranging from 2 to 29 years—for how long former smokers need to remain smoke-free before their risk approaches that of never-smokers.
- As smoking patterns shift toward low-intensity smoking in many populations, with more people smoking fewer cigarettes per day, there is a need to understand the cardiovascular risks of low-intensity smoking and the long-term benefits of quitting.

**What did the researchers do and find?:**

- We analyzed data from 22 prospective cohort studies with 323,826 adults (76% women), following participants for up to 19.9 years and documenting over 125,000 deaths and 54,000 cardiovascular events.
- Low-intensity smoking (2–5 cigarettes per day) was associated with a 50% higher risk of cardiovascular disease and a 60% higher risk of all-cause mortality compared to never smoking.
- The most substantial risk reduction across all outcomes occurred within the first decade following smoking cessation. However, continued risk diminution persisted beyond this initial period.

**What do these findings mean?:**

- Even occasional or low-intensity smoking significantly increases cardiovascular and mortality risks.
- The primary public health message of our paper is the importance of complete smoking cessation at younger ages
- Smoking cessation is associated with substantial risk reduction that begins immediately upon quitting and continues with a steep decline in risk during the first 10 years following smoking cessation.
- Limitations include the possibility that smoking habits changed over time since we only measured smoking at the study’s start, which may have led us to underestimate the true health risks. Moreover, we lacked information about other tobacco products like e-cigarettes, preventing us from studying people who use multiple tobacco products.

## Abstract 3

Using data from 22 prospective cohort studies, Erfan Tasdighi and colleagues investigate the relationships among smoking burden, intensity, and cessation duration across multiple cardiovascular outcomes.

## Introduction

Tobacco use is the leading preventable cause of cardiovascular disease (CVD) and mortality globally, contributing to more than 8 million deaths annually worldwide [1–3]. The relationship between tobacco smoking and cardiovascular disease has been firmly established through decades of landmark epidemiological research. Large-scale prospective investigations have consistently documented the profound cardiovascular impact of tobacco use, with the Australian 45 and Up Study demonstrating that current smokers have at least double the risk of developing most major cardiovascular diseases across 36 different disease subtypes [4]. Meta-analytic evidence from the CHANCES consortium, analyzing 503,905 older adults across 25 cohorts, revealed that smoking advances cardiovascular mortality by more than five years, with current smokers exhibiting a summary hazard ratio (HR) of 2.07 compared to never smokers [5]. Sex-specific analyses have shown that women may be disproportionately affected, with women who smoke having a 25% higher relative risk of coronary heart disease (CHD) compared to men with equivalent tobacco exposure [6]. These foundational studies have established smoking as one of the most potent modifiable risk factors for cardiovascular disease, with clear dose-response relationships that persist across diverse populations and the entire spectrum of cardiovascular pathology.

Beyond these well-recognized cardiovascular outcomes, smoking also significantly increases the risk of cardiac arrhythmias, including a 31% increased risk of atrial fibrillation (AFib) and flutter and a 50% increased risk of paroxysmal tachycardia [7]. The recognition of arrhythmic complications as smoking-related outcomes is particularly important given emerging evidence that approximately 17% of excess mortality among current smokers may be due to diseases not yet formally established as smoking-attributable, suggesting that the cardiovascular burden of smoking, including arrhythmic disorders, may be substantially underestimated in current public health assessments [8]. Public education campaigns and stringent regulations over the past few decades have led to a significant reduction in the use of conventional cigarettes, especially in high-income countries [9,10]. Moreover, a significant number of adult cigarette smokers express a desire to discontinue smoking, indicating widespread recognition of smoking’s adverse effects and a potential willingness to embrace cessation efforts [11]. However, data suggest that smokers who do not quit may be shifting their cigarette use to lower levels of smoking. Accordingly, a prospective cohort study in the National Institutes of Health–AARP (formerly known as the American Association of Retired Persons) the percentage of daily smokers consuming fewer than 10 cigarettes per day (CPD) has increased from 16% to 27% between 2005 and 2014, and the proportion of those who do not smoke daily rose from 19% to 23% [12]. A previous study showed that the risk for CHD was reduced in individuals who smoked 1 CPD compared to those smoking 20 CPD [13]. However, data regarding the impact of low-intensity smoking on cardiovascular diseases and death have not been fully explored [13].

Further, while the prevalence of former smokers is increasing, there is inconsistent evidence about how long it takes for their risk levels to align with those of individuals who have never smoked. Research suggests that for individuals who have quit smoking, it can take anywhere from 2 to 29 years for their risk of cardiovascular outcomes, as well as specific and all-cause mortality, to return to the same level as those who have never smoked. However, a recent large-scale meta-analysis demonstrated that smoking cessation confers substantial mortality benefits at any age, with short-term cessation (<3 years) reducing excess death risk by 90%–95% in younger adults and averting approximately 5 years of life lost. Long-term cessation (≥10 years) nearly eliminated excess mortality risk, achieving survival rates comparable to never smokers and averting about 10 years of life lost. This temporal variability in risk reduction underscores the complexity of quantifying smoking cessation benefits and emphasizes the importance of individualized risk assessment approaches [14–19].

The primary objective of the current study was to leverage a large study population with detailed participant phenotyping to provide robust dose–response risk estimates of cardiovascular and mortality outcomes associated with smoking intensity and cessation duration compared to individuals who have never smoked. Additionally, we sought to elucidate the distinctive aspects of the relationships involving cumulative pack-years, CPD, duration of cessation, and the concurrent association between cessation duration and smoking pack-years across nine outcomes. This information could assist the tobacco control community in developing evidence-based public policies, regulations, and clinical practice guidelines.

## Methods

### Ethics statement

This study was approved by the Johns Hopkins School of Medicine Institutional Review Board (Approval number: IRB00226738; Date: 24 June 2020). Parent cohorts had participants sign informed consent, and were covered by local IRBs. The Johns Hopkins IRB committee granted a waiver regarding informed consent in this study because this study used only data previously collected under individual cohort-level IRBs.

### Study population

CCC-Tobacco, a working group of the Cross-Cohort Collaboration (CCC), has created a harmonized dataset from 22 prospective cohort studies, encompassing 322,782 participants, 21 from the U.S. and 1 cohort from Brazil. CCC-Tobacco comprises both traditional cardiovascular cohorts (*N* = 12) and noncardiovascular cohorts aimed at studying aging (*N* = 10). The rationale and design of the CCC-Tobacco project have been previously described in detail [20]. Descriptions of these cohorts and their baseline characteristics are shown in S1 and S2 Tables.

### Definition of exposures

Participants who reported smoking fewer than 100 cigarettes in their lifetimes were classified as never smokers. Conventional cigarette use was defined as a lifetime use of at least 100 cigarettes. Current conventional cigarette use was characterized by self-reported ongoing use at the time of the baseline examination on individual cohort questionnaires. The Characteristics of smoking parameters by cohorts are shown in S3 Table. Moreover, the timeline of baseline examination of each cohort has been laid out in S4 Table. Former use was defined as self-reported cessation of smoking conventional cigarettes.

Smoking burden and intensity were assessed using two metrics at baseline: pack-years and CPD. Both pack-years and CPD were evaluated as continuous variables (i.e., per 10 increase) and also as categorical variables. Pack-years were categorized into four groups (≤5, 6–10, 11–20, and >20 pack-years). CPD categories were set as (≤1, 2–5, 6–10, 11–15, 16–20, and >20 CPD). While we analyzed the risk estimates for pack-years separately in both former and current smokers, CPD by its nature was evaluated only among current smokers.

The CPD was collected as a continuous variable in most studies. However, if a study collected the CPD as a categorical variable, we harmonized this variable as a continuous variable. For instance, if a cohort measured the CPD as categories of 1–3 CPD, we use the average number of CPD (i.e., 2 CPD) for harmonization purposes.

For pack-years, the cohorts were collected as continuous variables, or we calculated the pack-years as a continuous variable using the duration of smoking and CPD.

For analysis, we utilized both continuous and pre-defined categories for CPD and pack-years. For instance, the categorical analyses used the predefined categories (≤1 CPD, >1 to 5 CPD, >5 to 10 CPD, etc.) The spline models presented in the figures treated CPD as a continuous variable.

Cessation duration among former smokers was defined at the baseline examination of each cohort based on the years since quitting smoking at the baseline examination of each cohort, determined by the self-reported age of quitting. We categorized cessation duration into four groups (≤10, 11–20, 21–30, and 31–40 years since quitting smoking).

### Cardiovascular and mortality outcomes

The selection of outcomes was based on those that were systematically collected and formally adjudicated across most of the participating cohorts. Moreover, we included various cardiovascular outcomes to provide comprehensive insights into the potential cardiovascular risks associated with conventional cigarette tobacco products.

A total of nine outcomes relevant to cardiovascular health were collected and harmonized in CCC-Tobacco: myocardial infarction (MI), stroke, heart failure (HF), AFib), CHD, cardiovascular disease (CVD), CHD mortality, CVD mortality, and all-cause mortality. CHD events were defined as a composite of MI, coronary revascularization, or coronary death. CVD events were defined as a composite of all Atherosclerotic CVD events, including CHD, stroke, or cardiovascular death (coronary death, stroke death, other atherosclerotic death, or other CVD death).

Median follow-up by outcome, ranging from 8.7 years (AFib) to approximately 19 years for CHD (19.3), CVD (18.6), and mortality outcomes (19.4–19.9), underscoring the prolonged interval between baseline smoking exposure and observed events.

Cardiovascular outcomes in this study were harmonized across the 22 cohorts by using each cohort’s specific definitions of the outcomes, which in most cases were adjudicated by a dedicated adjudication committee.

In the occasion that an individual cohort had some, but not all, components (i.e., angina) of the CHD or CVD composite events, the components that were present were retained to represent a modified CHD or CVD composite for that cohort.

### Harmonization of covariates

The definitions of all demographics, anthropometric, and traditional risk factors, including hypertension, diabetes, hyperlipidemia, and dyslipidemia, have been previously described [20]. Data harmonization in CCC-Tobacco adhered to published best practices in the field and has been coded into a master file to enable replication.

Harmonization of covariates for race/ethnicity was based on self-reported data and adhered to the data collection protocols of each cohort into American Indian or Alaskan, Asian, Black/African American, Hispanic, White, and Other. Participants with missing information on race/ethnicity were categorized as “other” in this study.

In the case of missing supportive risk factor data in <10% of total participants, multiple imputations were conducted using the remaining non-missing risk factors within each individual cohort [21]. For missing continuous data related to diastolic blood pressure measurements, rule-based imputation was used, utilizing non-missing systolic blood pressure and binary risk factor data from the rest of the dataset, along with average diastolic blood pressure from comparable subgroups in the CCC-tobacco dataset. Missing data on blood pressure-lowering and lipid-lowering medication and hyperlipidemia were imputed based on age, gender, hypertension, diabetes, and presence of CHD at baseline. We use hyperlipidemia to impute blood pressure and lipid-lowering medication as well. Imputations were done based on the records with at most one missing risk factor, except for lipid-lowering medication in the SOF cohort, which allowed for at most two risk factors. Within the CCC-Tobacco dataset, these methods produce nearly identical mean and median values for all risk factors.

### Statistical analysis

These baseline characteristics are presented by baseline smoking status (conventional cigarette smoking: never, former, current). Age- and sex- adjusted absolute risk of each outcome in the total population and according to smoking status was based on the 2000 U.S. Census data.

Baseline variables were utilized to conduct adjusted Cox proportional hazards models in the pooled cohort to evaluate the association between smoking and the 9 study outcomes. The first multivariable model was adjusted for age, sex, race, and ethnicity (White, Black/African American, Asian, Hispanic, American Indian or Alaska Native, and Other), and education status (did not complete high school or less than 12 years of full-time education, completed high school or 12 years of full-time education, college degree or higher, or more than 12 years of full-time education). The second multivariable model was additionally adjusted for harmonized covariates, including body mass index (BMI), systolic blood pressure, diastolic blood pressure, diabetes, hyperlipidemia, antihypertensive medication use, lipid-lowering medication use, history of CHD at baseline, and any alcohol use. The reference group for all analyses was individuals who had never smoked. Of note, to consider the intra-group correlation within cohorts, we incorporated a shared frailty component into our Cox model, represented by the variable “cohort”, which includes 22 unique cohort identifiers. This approach enhances the robustness of our inference by acknowledging and adjusting for the nonindependence of survival times within cohorts, thereby providing a more accurate estimation of the association of covariates on survival.

Cubic splines for pack-years and CPD were utilized to allow for nonlinear associations between these continuous predictors and the HR of the 9 outcomes and these continuous predictors. The cubic splines were included in the Cox proportional hazards models, adjusting for age, sex, education, and cohort status.

Using a prediction model, we also evaluated the joint association of the cessation duration and smoking pack-years among former smokers. The Cox model’s predictions were used to compute the HR for each graded combination of cessation duration and smoking pack-years strata. The final heat plots illustrate the relative hazards across varying pack years and cessation periods, with the lines connecting areas of equivalent risk, providing insights into smoking cessation’s long-term effects and the risk of smoking intensity.

To examine temporal trends in smoking-related cardiovascular risks, we stratified participating cohorts by enrollment year using 2001 as the median cutpoint, comparing earlier (≤2,001) versus later (>2,001) enrollment periods. The WHI cohort was excluded from this analysis due to its exceptionally large sample size (*n* = 161,808), uniform enrollment year, and inclusion of only women, which created an analytical artifact that obscured true chronological trends in smoking epidemiology. This stratification approach allowed for balanced temporal comparison and assessment of how smoking-related cardiovascular HRss have evolved over time. Sensitivity analyses examining standardized rates by time period and censoring follow-up at 6 and 10 years produced results identical to the primary analysis using complete follow-up, confirming the robustness of our temporal trend findings. To address potential bias from the “sick quitter effect,” where individuals may be more likely to quit smoking after developing health conditions, additional methodological approaches were incorporated into the analysis. First, we leveraged the exclusion criteria of the cohort studies, which typically eliminated participants with pre-existing serious conditions (cancer, advanced kidney disease, nursing home residence, or other conditions preventing long-term participation). Second, we conducted sensitivity analyses reclassifying recent quitters (≤2 years) as current smokers to account for illness-induced smoking cessation and excluded participants with CHD at baseline. Third, we performed age-stratified analyses comparing participants younger than or equal to 60 years versus those older than 60 years to examine whether the relationships between smoking cessation and outcomes differed by age groups, as the “sick quitter effect” tends to be more pronounced in older populations.

This study was conducted according to a prospective analysis plan developed as part of the CCC framework. Due to the multi-cohort nature of this collaboration, each participating cohort received a slightly differently formatted proposal tailored to their specific data structure and governance requirements, though all followed the same core analytical approach and objectives. The primary statistical analysis plan was established prior to data analysis and focused on examining associations between smoking status, intensity, and cessation duration with cardiovascular and mortality outcomes.

Several modifications to the original analysis plan were made during the peer review process to strengthen the study’s methodological rigor. Specifically, the examination of temporal trends stratified by enrollment year was added in response to reviewer comments requesting an investigation of how smoking-related risks have evolved over time. The exclusion of the Women’s Health Initiative cohort from temporal trend analyses was implemented after identifying that its large sample size and uniform enrollment year created analytical artifacts that obscured true chronological trends. Additionally, methodological approaches to address the “sick quitter effect” were enhanced during peer review.

This study is reported as per the Strengthening the Reporting of Observational Studies in Epidemiology (STROBE) guideline (S1 STROBE Checklist).

## Results

The total analytic sample size was 323,826, with a mean (SD) age of 59.7 (11.9) years; 76% were women. A total of 46,125 (14.08%) individuals reported current smoking, 119,049 (36.4%) were never smokers, and 158,652 (49%) were former smokers of conventional cigarettes.

In general, compared to never users of conventional cigarettes, former smokers were older, and the prevalence of women was lower among current smokers. White and Black or African American race/ethnicities were the most prevalent among all smoking status groups. Never and former smokers had higher educational attainment compared to current smokers. Alcohol use was more prevalent among current and former smokers compared to never users. The current smokers showed a more adverse cardiometabolic profile compared to other groups (Table 1).

**Table 1:** Baseline characteristics across conventional cigarette use status.

	Never *N* = 158,688 (49%)	Former *N* = 119,049 (37%)	Current *N* = 46,125 (14%)
**Age, Mean (SD)**	59.81 (12.44)	62.05 (10.01)	53.19 (12.1)
**Female *N* (%)**	131,291 (82.74)	88,896 (74.67)	25,791 (55.92)
**Race and ethnicity *N* (%)**			
** White**	110,388 (69.56)	92,450 (77.66)	30,101 (65.26)
** Black or African American**	27,765 (17.50)	17,442 (14.65)	10,013 (21.71)
** Asian**	4,306 (2.71)	1,387 (1.17)	276 (0.60)
** Hispanic or Latino**	11,918 (7.51)	4,377 (3.68)	3,640 (7.89)
** American Indian or Alaskan Native**	3,905 (2.46)	3,076 (2.58)	1841 (3.99)
** Other**	125 (0.08)	143 (0.12)	193 (0.42)
**Education *N* (%)**			
** High School not completed**	18,150 (11.44)	12,081 (10.15)	8,902 (19.30)
** High school completed**	36,034 (22.71)	24,013 (20.17)	13,008 (28.20)
** College degree**	102,242 (64.43)	81,723 (68.65)	23,036 (49.94)
Alcohol use^*^, ***N* (%)**	60,880 (38.36)	62,365 (52.39)	29,894 (64.81)
**BMI, kg/m**^**2**^, **Mean (SD)**	28.14 (5.94)	28.36 (5.82)	27.16 (5.43)
**Hypertension, *N* (%)**	61,875 (38.99)	50,429 (42.36)	20,013 (43.39)
**Systolic BP, mmHg, Mean (SD)**	127.43 (18.91)	128.36 (18.68)	128.1 (20.31)
**Diastolic BP, mmHg, Mean (SD)**	75.38 (10.37)	75.78 (10.72)	78.54 (13.41)
BP medication^*^ ***N* (%)**	34,718 (21.88)	28,852 (24.24)	8,988 (19.49)
**Diabetes *N* (%)**	16,537 (10.42)	13,867 (11.65)	4,911 (10.65)
**Dyslipidemia *N* (%)**	48,362 (30.48)	37,933 (31.86)	22,680 (49.17)
**Hyperlipidemia *N* (%)**	30,594 (19.28)	25,140 (21.12)	14,723 (31.92)
**Lipid Lowering Medication *N* (%)**	17,689 (11.15)	17,095 (14.36)	3,821 (8.28)
**Total Cholesterol, mg/dL, Mean (SD)**	202.93 (42.99)	204.16 (43.38)	211.23 (45.7)
**LDL-C**^**4**^, **mg/dL, Mean (SD)**	123.19 (36.62)	124.05 (38)	131.32 (40.77)
**HDL-C, mg/dL, Mean (SD)**	53.31 (15.39)	51.27 (15.86)	48.01 (15.55)
**Triglyceride, mg/dL** ^*^	106 (106-153)	118 (84-118)	122 (122-179)
**Smoking intensity and cessation time**			
**Pack-Years** ^*^	NA	13 (3.5 - 30)	25 (10.9–45)
**Cigarettes per day**	NA	NA	19.8 (15.14)
**Cessation Duration (Years since quitting)**	NA	20.8 (13.1)	NA

Continuous variables are presented as means (standard deviations), and categorical variables are presented as percentages.

* Triglyceride and pack-years are presented as median (25,75 IQR)

BMI, body mass index; BP, blood pressure; HTG, hypertriglyceridemia; LDL-C, low-density lipoprotein cholesterol; HDL-C, high-density lipoprotein cholesterol

The median (25–75 IQR) smoking pack-years among former and current smokers was 13 (3.5–30) and 25 (10.9–45), respectively. The mean (SD) CPD amongst current smokers was 19.8 (15.14). The mean (SD) cessation duration among former smokers was 20.8 (13.1) years since quitting (Table 1).

The total number of events, age-adjusted incidence rate, and HR for each of the nine study outcomes were shown separately in men and women in Tables 2 and 3, respectively. In men, after full adjustment in Model 2, current male smokers exhibited significantly elevated risks with HRs of 1.70 (95% CI [1.58,1.82]) for MI, 1.55 (95% CI [1.41,1.71]) for stroke, 1.70 (95% CI [1.60,1.81]) for CHD, 1.74 (95% CI [1.66,1.83]) for CVD, 1.89 (95% CI [1.75,2.04]) for HF, 1.57 (95% CI [1.45,1.70]) for AFib, 1.87 (95% CI [1.71,2.04]) for CHD mortality, 1.87 (95% CI [1.75,2.00]) for CVD mortality, and 2.17 (95% CI [2.09,2.25]) for all-cause mortality (all *p* < 0.001) (Table 2). Women current smokers, compared with never smokers counterparts, demonstrated HRs of 2.17 (95% CI [2.05,2.30]) for MI, 1.77 (95% CI [1.67,1.87]) for stroke, 2.23 (95% CI [2.13,2.33]) for CHD, 2.07 (95% CI [2.00,2.14]) for CVD, 2.09 (95% CI [1.97,2.22]) for HF, 1.54 (95% CI [1.42,1.66]) for AFib, 2.48 (95% CI [2.33,2.63]) for CHD mortality, 2.32 (95% CI [2.22,2.42]) for CVD mortality, and 2.43 (95% CI [2.38,2.48]) for all-cause mortality (all *p* < 0.001). Former smokers in both sexes showed intermediate risk profiles, with incidence rates consistently falling between never and current smokers, and statistically significant HRs (all *p* < 0.001) typically ranging from 4% to 27% increased risk across outcomes after full adjustment in Model 2 (Table 3). Additional sensitivity analyses were conducted to assess the robustness of our findings. These included analyses excluding participants with reported CHD at baseline and stratifying participants by age (≤60 years versus >60 years), all of which yielded comparable results to the primary analysis. (S5–S7 Tables).

**Table 2:** Association between smoking status and incidence of cardiovascular and mortality outcomes among men.

Outcome	Never	Former	Current
**MI**			
** No. of Events**	1 679	2 585	2 074
** Age-adjusted incidence rate per 1,000 person-years**	4.92	6.44	9.41
** Unadjusted HR (95% CI)**	1.00	1.48 (1.39, 1.58)	1.73 (1.63, 1.85)
** Model 1 HR (95% CI)**	1.00	1.18 (1.10, 1.25)	1.65 (1.53, 1.76)
** Model 2 HR (95% CI)**	1.00	1.13 (1.06, 1.20)	1.70 (1.58, 1.82)
**Stroke**			
** No. of events**	1 178	1 665	953
** Age-adjusted incidence rate per 1,000 person-years**	3.37	3.70	4.41
** Unadjusted HR (95% CI)**	1.00	1.35 (1.25, 1.45)	1.11 (1.02, 1.21)
** Model 1 HR (95% CI)**	1.00	1.05 (0.98, 1.14)	1.49 (1.36, 1.63)
** Model 2 HR (95% CI)**	1.00	1.04 (0.96, 1.12)	1.55 (1.41, 1.71)
**CHD**			
** No. of events**	2 505	4 086	2 778
** Age-adjusted incidence rate per 1,000 person-years**	5.40	6.54	10.31
** Unadjusted HR (95% CI)**	1.00	1.51 (1.44, 1.59)	1.51 (1.43, 1.59)
** Model 1 HR (95% CI)**	1.00	1.17 (1.11, 1.23)	1.64 (1.55, 1.74)
** Model 2 HR (95% CI)**	1.00	1.11 (1.05, 1.17)	1.70 (1.60, 1.81)
**CVD**			
** No. of events**	4 202	6 624	3 928
** Age-adjusted incidence rate per 1,000 person-years**	10.03	12.18	16.81
** Unadjusted HR (95% CI)**	1.00	1.48 (1.42, 1.54)	1.29 (1.24, 1.35)
** Model 1 HR (95% CI)**	1.00	1.13 (1.09, 1.18)	1.66 (1.58, 1.74)
** Model 2 HR (95% CI)**	1.00	1.08 (1.04, 1.13)	1.74 (1.66, 1.83)
**Heart failure**			
** No. of events**	1 785	3 035	1 544
** Age-adjusted incidence rate per 1,000 person-years**	5.25	6.83	8.04
** Unadjusted HR (95% CI)**	1.00	1.69 (1.59, 1.79)	1.31 (1.22, 1.40)
** Model 1 HR (95% CI)**	1.00	1.21 (1.14, 1.28)	1.69 (1.57, 1.81)
** Model 2 HR (95% CI)**	1.00	1.15 (1.08, 1.22)	1.89 (1.75, 2.04)
**Atrial fibrillation**			
** No. of events**	1 815	2 740	1 247
** Age-adjusted incidence rate per 1,000 person-years**	8.23	10.38	10.79
** Unadjusted HR (95% CI)**	1.00	1.64 (1.55, 1.74)	1.07 (0.99, 1.15)
** Model 1 HR (95% CI)**	1.00	1.15 (1.08, 1.22)	1.48 (1.37, 1.60)
** Model 2 HR (95% CI)**	1.00	1.10 (1.03, 1.17)	1.57 (1.45, 1.70)
**CHD mortality**			
** No. of events**	1 261	2 161	1 337
** Age-adjusted incidence rate per 1,000 person-year**	2.85	3.62	5.58
** Unadjusted HR (95% CI)**	1.00	1.60 (1.50, 1.72)	1.42 (1.32, 1.54)
** Model 1 HR (95% CI)**	1.00	1.17 (1.09, 1.26)	1.76 (1.62, 1.92)
** Model 2 HR (95% CI)**	1.00	1.08 (1.00, 1.16)	1.87 (1.71, 2.04)
**CVD mortality**			
** No. of events**	2 386	3 908	2 084
** Age-adjusted incidence rate per 1,000 person-year**	5.63	6.59	9.23
** Unadjusted HR (95% CI)**	1.00	1.50 (1.43, 1.58)	1.14 (1.08, 1.21)
** Model 1 HR (95% CI)**	1.00	1.13 (1.08, 1.19)	1.76 (1.65, 1.88)
** Model 2 HR (95% CI)**	1.00	1.07 (1.01, 1.13)	1.87 (1.75, 2.00)
**All-cause mortality**			
** No. of events**	7 242	12 245	6 519
** Age-adjusted incidence rate per 1,000 person-year**	16.34	20.31	29.53
** Unadjusted HR (95% CI)**	1.00	1.64 (1.59, 1.69)	1.21 (1.17, 1.25)
** Model 1 HR (95% CI)**	1.00	1.21 (1.18, 1.25)	2.09 (2.01, 2.17)
** Model 2 HR (95% CI)**	1.00	1.19 (1.16, 1.23)	2.17 (2.09, 2.25)

Age-adjusted incidence rates were calculated according to the U.S. population standard for the year 2000.

Model 1 adjusted for age, sex, race and ethnicity, and education status.

Model 2 adjusted for age, sex, race and ethnicity, education status, body mass index, diabetes, hyperlipidemia, antihypertensive and lipid-lowering medication use, systolic blood pressure, diastolic blood pressure, history of coronary heart disease at baseline, and alcohol use.

Models include a shared frailty component for ‘cohort’ to account for intra-group correlation within the 22 unique cohorts.

MI, myocardial infarction; CHD, coronary heart disease; CVD, cardiovascular disease.

**Table 3:** Association between smoking Status and Incidence of cardiovascular and mortality outcomes among women.

Outcome	Never	Former	Current
**MI**			
** No. of events**	5 195	3 929	1 922
** Age-adjusted incidence rate per 1,000 person-year**	1.95	2.09	4.52
** Unadjusted HR (95% CI)**	1.00	1.07 (1.03, 1.12)	1.82 (1.73, 1.92)
** Model 1 HR (95% CI)**	1.00	1.19 (1.14, 1.24)	1.94 (1.83, 2.05)
** Model 2 HR (95% CI)**	1.00	1.21 (1.16, 1.26)	2.17 (2.05, 2.30)
**Stroke**			
** No. of events**	6 571	5 030	1 772
** Age-adjusted incidence rate per 1,000 person-year**	2.29	2.43	3.96
** Unadjusted HR (95% CI)**	1.00	1.09 (1.05, 1.13)	1.32 (1.25, 1.39)
** Model 1 HR (95% CI)**	1.00	1.14 (1.10, 1.19)	1.58 (1.49, 1.67)
** Model 2 HR (95% CI)**	1.00	1.16 (1.12, 1.20)	1.77 (1.67, 1.87)
**CHD**			
** No. of events**	10 237	7 735	2 982
** Age-adjusted incidence rate per 1,000 person-year**	2.74	2.89	5.69
** Unadjusted HR (95% CI)**	1.00	1.07 (1.04, 1.10)	1.55 (1.49, 1.62)
** Model 1 HR (95% CI)**	1.00	1.18 (1.14, 1.21)	1.97 (1.89, 2.05)
** Model 2 HR (95% CI)**	1.00	1.20 (1.16, 1.23)	2.23 (2.13, 2.33)
**CVD**			
** No. of events**	19 572	14 253	5 058
** Age-adjusted incidence rate per 1,000 person-year**	5.28	5.21	10.05
** Unadjusted HR (95% CI)**	1.00	1.04 (1.02, 1.06)	1.37 (1.33, 1.42)
** Model 1 HR (95% CI)**	1.00	1.14 (1.12, 1.17)	1.83 (1.77, 1.89)
** Model 2 HR (95% CI)**	1.00	1.16 (1.14, 1.19)	2.07 (2.00, 2.14)
**Heart failure**			
** No. of events**	5 005	3 470	1 917
** Age-adjusted incidence rate per 1,000 person-year**	1.86	1.63	4.00
** Unadjusted HR (95% CI)**	1.00	1.00 (0.95, 1.04)	1.69 (1.60, 1.78)
** Model 1 HR (95% CI)**	1.00	1.24 (1.19, 1.30)	1.71 (1.62, 1.81)
** Model 2 HR (95% CI)**	1.00	1.27 (1.21, 1.33)	2.09 (1.97, 2.22)
**Atrial fibrillation**			
** No. of events**	2 922	1 541	1 074
** Age-adjusted incidence rate per 1,000 person-year**	3.97	3.42	5.67
** Unadjusted HR (95% CI)**	1.00	1.13 (1.06, 1.20)	0.88 (0.82, 0.94)
** Model 1 HR (95% CI)**	1.00	1.15 (1.07, 1.22)	1.34 (1.24, 1.44)
** Model 2 HR (95% CI)**	1.00	1.15 (1.07, 1.22)	1.54 (1.42, 1.66)
**CHD mortality**			
** No. of events**	6 288	4 689	1 510
** Age-adjusted incidence rate per 1,000 person-year**	1.55	1.58	2.85
** Unadjusted HR (95% CI)**	1.00	1.05 (1.01, 1.09)	1.32 (1.24, 1.39)
** Model 1 HR (95% CI)**	1.00	1.18 (1.14, 1.23)	2.11 (1.99, 2.24)
** Model 2 HR (95% CI)**	1.00	1.21 (1.16, 1.26)	2.48 (2.33, 2.63)
**CVD mortality**			
** No. of events**	12 655	8 928	2 862
** Age-adjusted incidence rate per 1,000 person-year**	3.25	3.06	5.49
** Unadjusted HR (95% CI)**	1.00	0.99 (0.97, 1.02)	1.20 (1.15, 1.25)
** Model 1 HR (95% CI)**	1.00	1.14 (1.11, 1.18)	1.99 (1.91, 2.08)
** Model 2 HR (95% CI)**	1.00	1.17 (1.14, 1.20)	2.32 (2.22, 2.42)
**All-cause mortality**			
** No. of events**	48 015	37 643	12 305
** Age-adjusted incidence rate per 1,000 person-year**	12.17	13.10	22.55
** Unadjusted HR (95% CI)**	1.00	1.12 (1.11, 1.14)	1.36 (1.33, 1.38)
** Model 1 HR (95% CI)**	1.00	1.21 (1.19, 1.23)	2.23 (2.18, 2.27)
** Model 2 HR (95% CI)**	1.00	1.23 (1.21, 1.24)	2.43 (2.38, 2.48)

Age-adjusted incidence rates were calculated according to the U.S. population standard for the year 2000.

Model 1 adjusted for age, sex, race and ethnicity, and education status.

Model 2 adjusted for age, sex, race and ethnicity, education status, body mass index, diabetes, hyperlipidemia, antihypertensive and lipid-lowering medication use, systolic blood pressure, diastolic blood pressure, history of coronary heart disease at baseline, and alcohol use.

Models include a shared frailty component for ‘cohort’ to account for intra-group correlation within the 22 unique cohorts.

MI, myocardial infarction; CHD, coronary heart disease; CVD, cardiovascular disease.

### Smoking intensity and cardiovascular and mortality outcomes

The association between continuous and categorical values of smoking pack-years and evaluated outcomes among former and current smokers compared to never smokers is presented in S8 and S9 Tables. For every 10 pack-year increments, there was a further increased risk of between 2.4% and 4.6% across the outcomes. Former smokers with up to 5 pack-years of smoking did not show a significant association with any of the outcomes. Compared with never-smokers, current smokers with ≤5 pack-years showed an increased risk for all the outcomes except for AFib. There was an increased risk for all the outcomes for current smokers with >6 pack-years.

**Fig 1:**
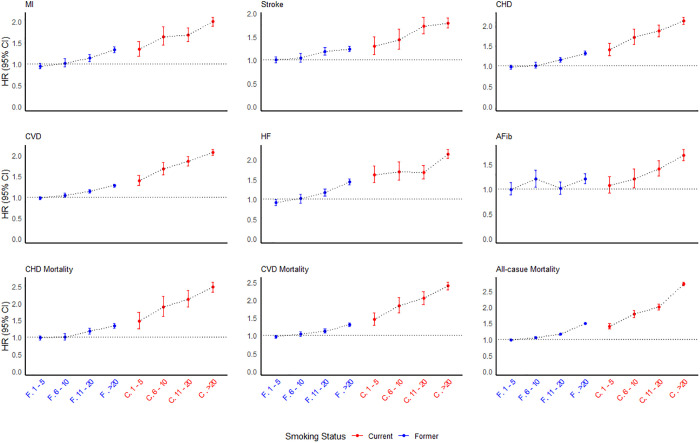
Graded association between pack-year groups in former and current smokers and cardiovascular and mortality outcomes. Groups have been created based on the smoking status (i.e., F: former smokers, C: current smokers) and the pack year count category. The hazard ratio (HR) and 95% CI have been revived from the comprehensive adjusted model. The Cox models were adjusted for age, sex, race and ethnicity, education status, cohort, body mass index (BMI), systolic blood pressure, diastolic blood pressure, diabetes, hyperlipidemia, antihypertensive medication use, lipid-lowering medication use, history of coronary heart disease at baseline and any alcohol use.

Fig 1 illustrates the HRs (95% CI) of each pack-year category and cardiovascular and mortality outcomes for former and current smokers compared to never smokers. This figure shows that current smokers consistently had a higher risk than former smokers, irrespective of the pack-year category, for all the outcomes except for AFib.

**Fig 2:**
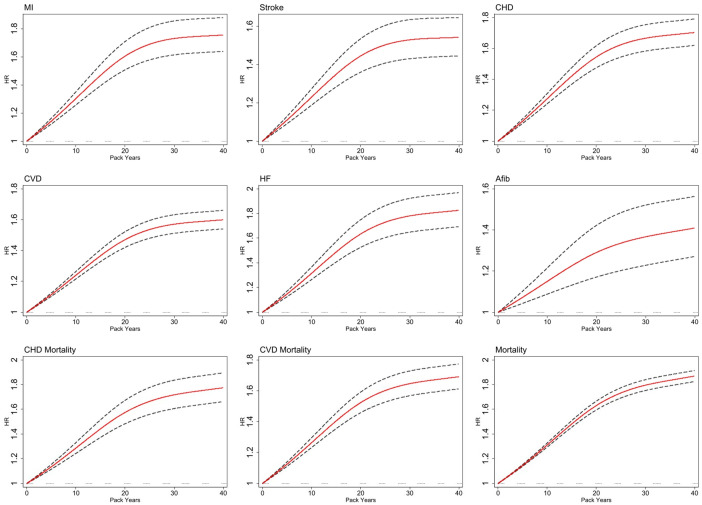
Association between pack years of smoking and hazard ratio (HR) for CVD and mortality outcomes: a cubic spline analysis. MI, myocardial infarction; CHD, coronary heart disease; CVD, cardiovascular disease; HF, heart failure; AFib, atrial fibrillation; 95% confidence interval has been shown with dashed line.

The cubic splines analysis (illustrated in Fig 2) revealed a nonlinear dose-response relationship between pack-years of smoking and the risk of cardiovascular and mortality outcomes. Initially, the curve began with a steep ascent, indicating that the initial 20 pack years were associated with a pronounced increase in evaluated outcomes. After approximately 20 pack years, the curves still increased but at a decelerating rate.

**Fig 3:**
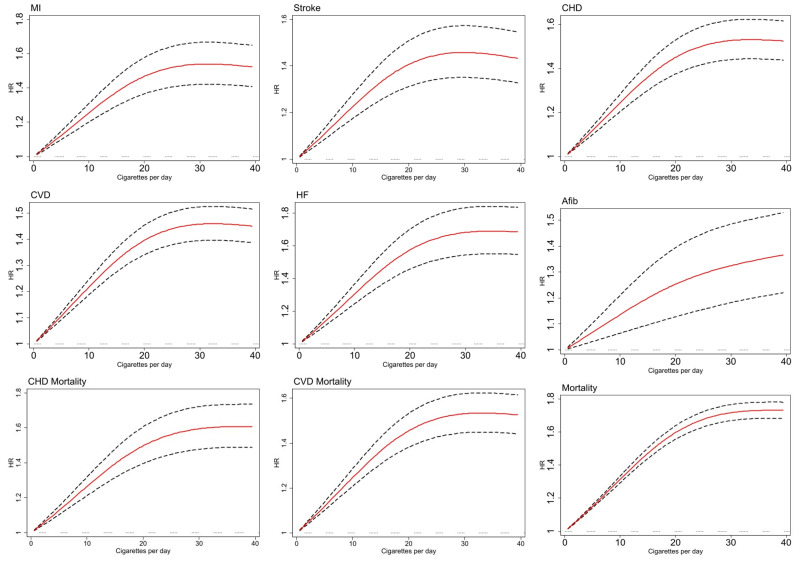
Relationship between cigarettes per day and hazard ratio (HR) for CVD and mortality outcomes: a cubic spline model evaluation. MI, myocardial infarction; CHD, coronary heart disease; CVD, cardiovascular disease; HF, heart failure; AFib, atrial fibrillation; 95% confidence interval has been shown with a dashed line.

The association between continuous and categorical values of CPD and evaluated outcomes among current smokers is presented in Table 4. Participants with CPD ≤1 showed significantly increased risks across most cardiovascular outcomes, with HRs ranging from 1.16 (95% CI [0.87,1.53]; *p* = 0.30) for AFib to 2.07 (95% CI [1.69,2.54]; *p* < 0.001) for HF. Those smoking 2–5 CPD demonstrated consistently elevated risks compared to never-smokers, with HRs ranging from 1.26 (95% CI [1.09,1.45]; *p* = 0.002) for AFib to 1.60 (95% CI [1.52,1.69]; *p* < 0.001) for all-cause mortality, indicating substantial cardiovascular harm even at very low levels of smoking exposure. Of note, compared to other outcomes, AFib estimates showed wider confidence intervals, which demonstrate more heterogeneity in the effects of smoking on AFib. Using Cox proportional hazards models with cubic spline functions indicated a significant risk increase associated with the first 20 CPD across all outcomes assessed. Beyond this point, the risk escalation began to level off for most outcomes. However, the risk associated with AFib continued to climb even after exceeding 20 CPD, diverging from the pattern seen with other conditions (Fig 3 and S10 Table).

**Table 4:** Association between the number of cigarettes per day with cardiovascular outcomes among current cigarette users.

	Per 10 increments of CPD^a^	CPD categories						
		Never-smokers [Reference]^b^	≤ 1 *N* = 1,423	2–5 *N* = 4,864	6–10 *N* = 8,552	11–15 *N* = 2,779	16–20 *N* = 14,032	> 20 *N* = 11,826
**CV outcomes**								
**MI**								
Unadjusted HR (95% CI)	**1.112 (1.098, 1.127)**		1.22 (0.99, 1.51)	**1.50 (1.34, 1.67)**	**1.65 (1.52, 1.79)**	**1.90 (1.67, 2.17)**	**2.31 (2.18, 2.45)**	**2.91 (2.74, 3.10)**
Model 1 HR (95% CI)	**1.063 (1.041, 1.085)**	**1**	1.23 (0.99, 1.52)	**1.43 (1.28, 1.60)**	**1.67 (1.54, 1.82)**	**1.53 (1.34, 1.75)**	**1.91 (1.79, 2.03)**	**2.03 (1.89, 2.18)**
Model 2 HR (95% CI)	**1.065 (1.042, 1.088)**	**1**	**1.42 (1.13, 1.77)**	**1.50 (1.32, 1.70)**	**1.91 (1.75, 2.08)**	**1.74 (1.51, 2.02)**	**2.07 (1.94, 2.21)**	**2.20 (2.04, 2.38)**
**Stroke**								
Unadjusted HR (95% CI)	**0.997 (0.970, 1.026)**		0.91 (0.72, 1.14)	1.08 (0.96, 1.22)	**1.32 (1.21, 1.43)**	**1.17 (1.00, 1.36)**	**1.40 (1.31, 1.49)**	**1.14 (1.04, 1.24)**
Model 1 HR (95% CI)	**1.045 (1.017, 1.074)**	**1**	1.18 (0.94, 1.49)	**1.28 (1.13, 1.45)**	**1.52 (1.40, 1.66)**	**1.40 (1.19, 1.64)**	**1.64 (1.53, 1.76)**	**1.64 (1.49, 1.79)**
Model 2 HR (95% CI)	**1.058 (1.029, 1.089)**	**1**	1.26 (0.98, 1.63)	**1.41 (1.23, 1.61)**	**1.67 (1.52, 1.83)**	**1.69 (1.42, 1.99)**	**1.83 (1.70, 1.97)**	**1.82 (1.65, 2.00)**
**CHD**								
Unadjusted HR (95% CI)	**1.072 (1.056, 1.088)**		1.06 (0.89, 1.25)	**1.33 (1.21, 1.45)**	**1.49 (1.40, 1.59)**	**1.69 (1.51, 1.88)**	**1.96 (1.87, 2.06)**	**1.99 (1.89, 2.10)**
Model 1 HR (95% CI)	**1.060 (1.041, 1.079)**	**1**	**1.24 (1.04, 1.47)**	**1.41 (1.29, 1.55)**	**1.68 (1.57, 1.79)**	**1.65 (1.48, 1.85)**	**1.99 (1.89, 2.09)**	**2.12 (1.99, 2.25)**
Model 2 HR (95% CI)	**1.062 (1.042, 1.082)**	**1**	**1.48 (1.24, 1.78)**	**1.51 (1.36, 1.67)**	**1.94 (1.81, 2.08)**	**1.91 (1.69, 2.16)**	**2.18 (2.07, 2.30)**	**2.32 (2.18, 2.48)**
**CVD**								
Unadjusted HR (95% CI)	**1.044 (1.030, 1.058)**		0.93 (0.82, 1.07)	**1.16 (1.08, 1.24)**	**1.31 (1.25, 1.38)**	**1.46 (1.34, 1.59)**	**1.68 (1.62, 1.74)**	**1.52 (1.46, 1.59)**
Model 1 HR (95% CI)	**1.051 (1.036, 1.066)**	**1**	**1.26 (1.10, 1.44)**	**1.39 (1.29, 1.49)**	**1.61 (1.54, 1.70)**	**1.62 (1.48, 1.76)**	**1.93 (1.85, 2.00)**	**2.02 (1.92, 2.12)**
Model 2 HR (95% CI)	**1.057 (1.041, 1.074)**	**1**	**1.47 (1.28, 1.70)**	**1.50 (1.38, 1.62)**	**1.83 (1.73, 1.93)**	**1.87 (1.69, 2.06)**	**2.12 (2.03, 2.21)**	**2.23 (2.12, 2.35)**
**Heart failure**								
Unadjusted HR (95% CI)	**1.086 (1.065, 1.106)**		**1.34 (1.10, 1.63)**	**1.45 (1.30, 1.62)**	**1.57 (1.45, 1.70)**	**1.97 (1.73, 2.23)**	**1.95 (1.83, 2.07)**	**2.05 (1.90, 2.21)**
Model 1 HR (95% CI)	**1.057 (1.032, 1.082)**	**1**	**1.53 (1.25, 1.86)**	**1.33 (1.19, 1.48)**	**1.57 (1.44, 1.70)**	**1.59 (1.41, 1.81)**	**1.82 (1.71, 1.94)**	**1.91 (1.76, 2.06)**
Model 2 HR (95% CI)	**1.052 (1.026, 1.078)**	**1**	**2.07 (1.69, 2.54)**	**1.57 (1.39, 1.78)**	**1.90 (1.74, 2.08)**	**1.98 (1.72, 2.27)**	**2.22 (2.08, 2.38)**	**2.22 (2.04, 2.42)**
**AFib**								
Unadjusted HR (95% CI)	**1.089 (1.064, 1.115)**		0.68 (0.52, 0.89)	0.81 (0.71, 0.94)	0.93 (0.83, 1.03)	0.97 (0.83, 1.12)	1.03 (0.95, 1.12)	**1.18 (1.08, 1.28)**
Model 1 HR (95% CI)	**1.073 (1.040, 1.108)**	**1**	1.01 (0.77, 1.32)	1.12 (0.97, 1.29)	**1.36 (1.22, 1.52)**	**1.37 (1.18, 1.59)**	**1.44 (1.32, 1.56)**	**1.64 (1.49, 1.79)**
Model 2 HR (95% CI)	**1.069 (1.038, 1.102)**	**1**	1.16 (0.87, 1.53)	**1.26 (1.09, 1.45)**	**1.54 (1.37, 1.72)**	**1.64 (1.41, 1.91)**	**1.62 (1.49, 1.77)**	**1.81 (1.65, 2.00)**
**Mortality outcomes**								
**CHD mortality**								
Unadjusted HR (95% CI)	**1.055 (1.030, 1.080)**		1.00 (0.79, 1.25)	**1.15 (1.01, 1.30)**	**1.40 (1.28, 1.52)**	**1.49 (1.28, 1.74)**	**1.69 (1.58, 1.80)**	**1.61 (1.49, 1.74)**
Model 1 HR (95% CI)	**1.067 (1.039, 1.096)**	**1**	**1.37 (1.09, 1.73)**	**1.43 (1.26, 1.63)**	**1.80 (1.65, 1.96)**	**1.93 (1.65, 2.26)**	**2.32 (2.16, 2.49)**	**2.34 (2.14, 2.57)**
Model 2 HR (95% CI)	**1.079 (1.050, 1.110)**	**1**	**1.76 (1.38, 2.24)**	**1.55 (1.34, 1.79)**	**2.11 (1.92, 2.32)**	**2.38 (1.98, 2.85)**	**2.60 (2.41, 2.82)**	**2.68 (2.43, 2.96)**
**CVD mortality**								
Unadjusted HR (95% CI)	**1.042 (1.022, 1.062)**		0.83 (0.69, 0.98)	1.02 (0.93, 1.11)	**1.18 (1.11, 1.26)**	**1.28 (1.14, 1.44)**	**1.49 (1.42, 1.57)**	**1.31 (1.24, 1.40)**
Model 1 HR (95% CI)	**1.057 (1.036, 1.078)**	**1**	**1.32 (1.11, 1.57)**	**1.43 (1.30, 1.57)**	**1.72 (1.61, 1.84)**	**1.80 (1.60, 2.03)**	**2.22 (2.11, 2.34)**	**2.24 (2.09, 2.39)**
Model 2 HR (95% CI)	**1.070 (1.048, 1.092)**	**1**	**1.67 (1.39, 2.00)**	**1.57 (1.41, 1.75)**	**1.99 (1.85, 2.14)**	**2.06 (1.79, 2.37)**	**2.48 (2.34, 2.63)**	**2.54 (2.36, 2.74)**
**All-cause mortality**								
Unadjusted HR (95% CI)	**1.049 (1.039, 1.060)**		0.83 (0.76, 0.91)	1.01 (0.96, 1.06)	**1.28 (1.24, 1.33)**	**1.24 (1.17, 1.32)**	**1.57 (1.54, 1.61)**	**1.39 (1.35, 1.43)**
Model 1 HR (95% CI)	**1.095 (1.085, 1.105)**	**1**	**1.36 (1.24, 1.49)**	**1.53 (1.45, 1.60)**	**1.95 (1.89, 2.01)**	**2.09 (1.96, 2.22)**	**2.52 (2.45, 2.59)**	**2.94 (2.84, 3.04)**
Model 2 HR (95% CI)	**1.101 (1.091, 1.112)**	**1**	**1.51 (1.37, 1.67)**	**1.60 (1.52, 1.69)**	**2.14 (2.07, 2.22)**	**2.30 (2.14, 2.47)**	**2.75 (2.67, 2.83)**	**3.20 (3.08, 3.32)**

Model 1 adjusted for age, sex, race and ethnicity, and education status.

Model 2 adjusted for age, sex, race and ethnicity, education status, body mass index, diabetes, hyperlipidemia, antihypertensive and lipid-lowering medication use, systolic blood pressure, diastolic blood pressure, history of coronary heart disease at baseline, and alcohol use.

Models include a shared frailty component for ‘cohort’ to account for intra-group correlation within the 22 unique cohorts.

^a^Number of cigarettes per day was considered as a continuous variable.

^b^This is the reference group (i.e., never-smokers) for the categorical analysis.

CPD, cigarettes per day; MI, myocardial infarction; AFib, atrial fibrillation; CHD, coronary heart disease; CVD, cardiovascular disease.

A sensitivity analysis was performed to compare the association of smoking status and outcomes based on the individuals’ enrollment year. Among participants enrolled after 2001 compared to those enrolled in 2001 or earlier, current smokers demonstrated consistently higher HRs across all measured outcomes. In the adjusted Model 2, current smokers enrolled after 2001 showed markedly elevated risks for MI (HR 1.92, 95% CI [1.72,2.13] versus HR 1.74, 95% CI [1.64,1.85]), stroke (HR 1.70, 95% CI [1.50,1.93] versus HR 1.55, 95% CI [1.44,1.66]), CHD (HR 1.97, 95% CI [1.79,2.16] versus HR 1.73, 95% CI [1.65,1.82]), and overall mortality (HR 2.44, 95% CI [2.32,2.57] versus HR 2.05, 95% CI [1.99,2.11]; all *p* < 0.001). Former smokers also exhibited modestly increased risks in the later enrollment period for most cardiovascular outcomes, though the temporal differences were less pronounced than those observed for current smokers (Table 5).

**Table 5:** Association between smoking status and incidence of cardiovascular and mortality outcomes stratified by enrollment year.

Outcome	Enrolled before or in 2001		Enrolled after 2001	
	Former	Current	Former	Current
**MI**				
Unadjusted HR (95% CI)	1.37 (1.29, 1.45)	1.66 (1.57, 1.75)	1.77 (1.64, 1.91)	1.85 (1.67, 2.05)
Model 1 HR (95% CI)	1.12 (1.06, 1.19)	1.56 (1.47, 1.65)	1.28 (1.18, 1.39)	1.89 (1.70, 2.09)
Model 2 HR (95% CI)	1.14 (1.07, 1.21)	1.74 (1.64, 1.85)	1.23 (1.13, 1.33)	1.92 (1.72, 2.13)
**Stroke**				
Unadjusted HR (95% CI)	1.15 (1.08, 1.22)	0.97 (0.91, 1.04)	1.37 (1.26, 1.50)	1.56 (1.39, 1.75)
Model 1 HR (95% CI)	1.03 (0.97, 1.10)	1.39 (1.30, 1.49)	1.09 (0.99, 1.19)	1.70 (1.51, 1.92)
Model 2 HR (95% CI)	1.05 (0.99, 1.12)	1.55 (1.44, 1.66)	1.08 (0.98, 1.19)	1.70 (1.50, 1.93)
**CHD**				
Unadjusted HR (95% CI)	1.32 (1.26, 1.38)	1.36 (1.31, 1.43)	1.85 (1.73, 1.98)	1.95 (1.79, 2.13)
Model 1 HR (95% CI)	1.10 (1.05, 1.15)	1.54 (1.47, 1.62)	1.25 (1.17, 1.34)	1.93 (1.76, 2.10)
Model 2 HR (95% CI)	1.10 (1.05, 1.15)	1.73 (1.65, 1.82)	1.20 (1.11, 1.28)	1.97 (1.79, 2.16)
**CVD**				
Unadjusted HR (95% CI)	1.25 (1.21, 1.29)	1.13 (1.09, 1.16)	1.68 (1.59, 1.76)	1.84 (1.72, 1.96)
Model 1 HR (95% CI)	1.08 (1.04, 1.11)	1.56 (1.50, 1.62)	1.18 (1.12, 1.24)	1.93 (1.81, 2.07)
Model 2 HR (95% CI)	1.08 (1.04, 1.12)	1.75 (1.69, 1.82)	1.14 (1.08, 1.21)	1.97 (1.83, 2.11)
**HF**				
Unadjusted HR (95% CI)	1.47 (1.40, 1.54)	1.16 (1.10, 1.22)	1.57 (1.47, 1.69)	1.51 (1.37, 1.67)
Model 1 HR (95% CI)	1.22 (1.16, 1.28)	1.64 (1.56, 1.74)	1.18 (1.10, 1.27)	1.60 (1.45, 1.77)
Model 2 HR (95% CI)	1.23 (1.17, 1.30)	2.01 (1.90, 2.13)	1.14 (1.06, 1.23)	1.79 (1.61, 1.98)
**AFib**				
Unadjusted HR (95% CI)	1.48 (1.41, 1.56)	0.95 (0.90, 1.00)	1.80 (1.64, 1.97)	1.23 (1.07, 1.42)
Model 1 HR (95% CI)	1.10 (1.05, 1.16)	1.42 (1.34, 1.50)	1.18 (1.08, 1.30)	1.31 (1.13, 1.52)
Model 2 HR (95% CI)	1.09 (1.04, 1.15)	1.59 (1.50, 1.69)	1.13 (1.03, 1.24)	1.40 (1.21, 1.62)
**CHD mortality**				
Unadjusted HR (95% CI)	1.34 (1.27, 1.42)	1.13 (1.06, 1.20)	1.93 (1.74, 2.15)	2.05 (1.79, 2.34)
Model 1 HR (95% CI)	1.09 (1.02, 1.15)	1.65 (1.54, 1.77)	1.27 (1.14, 1.42)	2.13 (1.85, 2.45)
Model 2 HR (95% CI)	1.06 (1.00, 1.13)	1.92 (1.79, 2.06)	1.21 (1.08, 1.35)	2.24 (1.93, 2.59)
**CVD mortality**				
Unadjusted HR (95% CI)	1.25 (1.20, 1.30)	0.95 (0.91, 0.99)	1.63 (1.51, 1.75)	1.74 (1.58, 1.91)
Model 1 HR (95% CI)	1.07 (1.03, 1.12)	1.65 (1.57, 1.74)	1.18 (1.10, 1.27)	2.05 (1.86, 2.27)
Model 2 HR (95% CI)	1.06 (1.02, 1.11)	1.90 (1.80, 2.00)	1.15 (1.07, 1.24)	2.09 (1.88, 2.33)
**All-cause mortality**				
Unadjusted HR (95% CI)	1.38 (1.35, 1.42)	1.06 (1.04, 1.09)	1.75 (1.69, 1.82)	1.99 (1.90, 2.09)
Model 1 HR (95% CI)	1.18 (1.15, 1.20)	1.89 (1.84, 1.94)	1.25 (1.20, 1.30)	2.35 (2.24, 2.47)
Model 2 HR (95% CI)	1.18 (1.15, 1.21)	2.05 (1.99, 2.11)	1.24 (1.19, 1.29)	2.44 (2.32, 2.57)

Model 1 adjusted for age, sex, race and ethnicity, and education status.

Model 2 adjusted for age, sex, race and ethnicity, education status, body mass index, diabetes, hyperlipidemia, antihypertensive and lipid-lowering medication use, systolic blood pressure, diastolic blood pressure, history of coronary heart disease at baseline, and alcohol use.

Models include a shared frailty component for “cohort” to account for intra-group correlation within the 22 unique cohorts.

MI, myocardial infarction; CHD, coronary heart disease; CVD, cardiovascular disease.

### Cessation duration and cardiovascular and mortality outcomes

**Fig 4:**
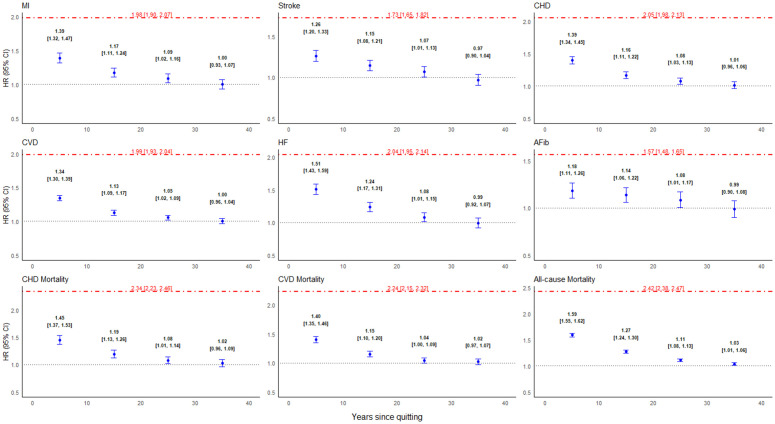
CVD and mortality outcomes hazard ratio (HR) by years since quit smoking among former smokers compared with current and never smokers. MI, myocardial infarction; CHD, coronary heart disease; CVD, cardiovascular disease, HF, heart failure, AFib, atrial fibrillation. Current smokers presented with a red dot-dashed line. The reference group is never-smokers presented with a dotted black line.

The association among former smokers between cessation duration categories and each evaluated outcome compared with never-smokers is illustrated in Fig 4. The higher risk for MI with HR 1.09 (95% CI [1.02,1.16]; *p* = 0.012), AFib with HR 1.08 (95% CI [1.01,1.17]; *p* = 0.049), and CVD mortality with HR 1.04 (95% CI [1.00,1.09]; *p* = 0.018) remained significant among former smokers with 21–30 years since quitting. The age-stratified analyses showed that both age groups demonstrated substantial benefits from smoking cessation across all cardiovascular outcomes and mortality endpoints. (S2 and S3 Figs). For most outcomes, risk normalization (HR approaching 1.0, similar to never smokers) occurred more rapidly in the younger age group. For instance, MI, stroke, and CHD HRs declined to near or below 1.0 by the 20–30 year mark for participants ≤60 years old.

**Fig 5:**
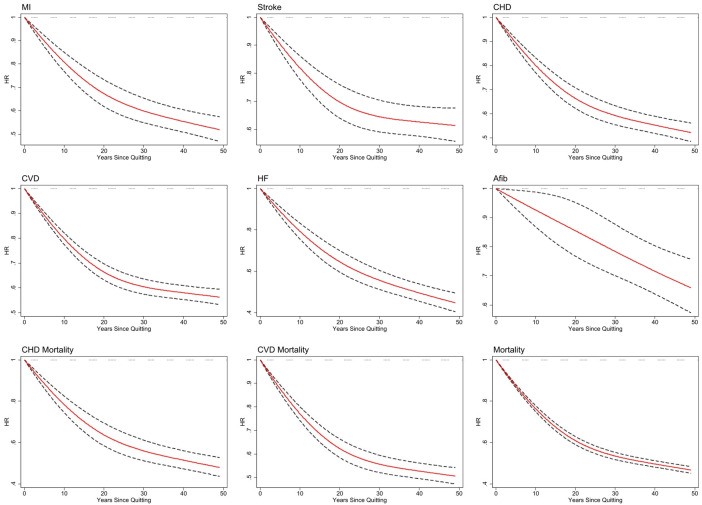
Cessation duration and hazard ratio (HR) for CVD and mortality outcomes: a cubic spline model evaluation. MI, myocardial infarction; CHD, coronary heart disease; CVD, cardiovascular disease; HF, heart failure; AFib, atrial fibrillation; 95% confidence interval has been shown with a dashed line.

Fig 5 illustrates the nonlinear relationship between the duration of cessation and the risk reduction for various outcomes. The curve demonstrates a more pronounced decrease in risk during the initial 15 years following cessation, transitioning to a plateau phase after approximately 20 years for most outcomes except for AFib, for which the risk continues to descend even beyond 40 years of cessation.

**Fig 6:**
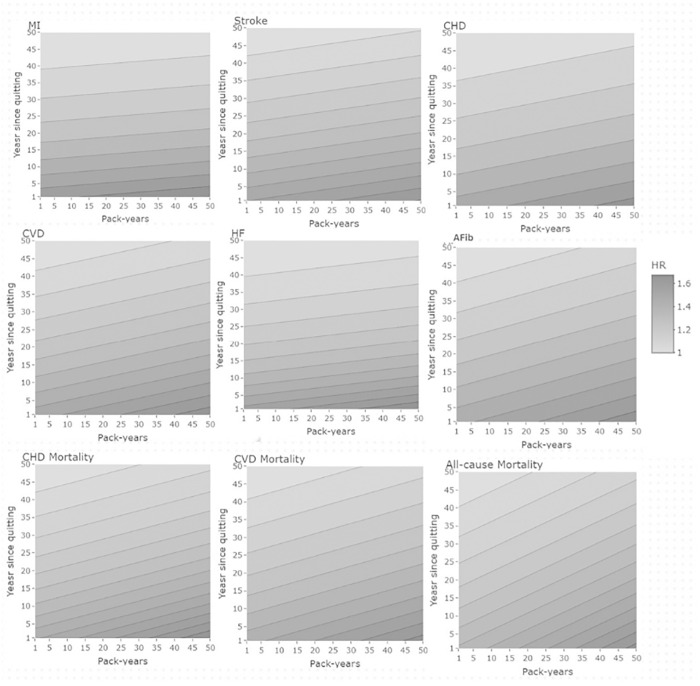
Cessation duration and pack-years concurrent effect on the hazard ratio (HR) for cardiovascular and mortality outcomes. MI, myocardial infarction; CHD, coronary heart disease; CVD, cardiovascular disease; HF, heart failure; AFib, atrial fibrillation.

Fig 6 depicts predicted HRs for each outcome based on concurrent consideration of smoking cessation duration and the number of pack-years among former smokers. Notably, for cardiovascular and mortality outcomes, the change in predicted HR (i.e., change in the color gradient) along the time since cessation y-axis exceeds the change in predicted HR across the cumulative pack-years x-axis. A visual analysis of the lines connecting areas of equivalent risk indicates that 5–10 additional years of time since smoking cessation may be roughly comparable to an incremental 30–50 pack-years of smoking burden in terms of total risk. Participants with the shortest time since cessation and with the largest number of pack-years were at the highest risk, while those with the longest time since cessation with the smallest number of pack-years were at the lowest risk.

Our sensitivity analyses examining the association between cessation duration and evaluated outcomes were conducted to assess potential confounding from the sick-quitter effect. Reclassification of recent quitters (≤2 years) as current smokers to account for illness-induced smoking cessation yielded results consistent with the primary analysis (S1 Fig). Additionally, age-stratified analyses were performed, given that the sick-quitter effect is more pronounced in older populations (S2 and S3 Figs), which confirmed the immediate risk reduction following cessation and demonstrated continued steep risk attenuation for at least 20 years post-cessation.

## Discussion

This comprehensive study, encompassing 22 predominantly U.S.-based cohorts with follow-up for cardiovascular outcomes spanning a median of 24 years, presents a large and detailed analysis of the impacts of different smoking measures on cardiovascular, cause-specific, and all-cause mortality outcomes. Our analysis delivers point estimates of risk for former and current smoking, with an emphasis on low-intensity smoking behaviors. In particular, we delineate the specific dynamics of the relationships between smoking pack-years, CPD, and cessation duration and cardiovascular and mortality outcomes. This study also highlights the complex interplay and nonlinear associations between cumulative smoking exposure and the time since smoking cessation in former smokers, underscoring the critical need to educate the public and promote early smoking cessation.

The findings of our research underscore the assertion that no level of smoking is without risk. Our findings indicate that current smokers using up to 1 CPD are associated with elevated risks of cardiovascular and mortality incidents, except for stroke and AFib. Notably, the 2–5 CPD range was associated with an increased risk for all assessed outcomes. Consistent with these findings, it has been reported that men and women smoking 3–5 CPD exhibited HRs for all-cause mortality of 45% increased risk (95% CI [33, 59]; *p* < 0.001) and 49% increased risk (95% CI [34, 66]; *p* < 0.001), respectively [22]. Additionally, another study investigating 505,500 nationally representative U.S. adults revealed that lifelong non-daily smokers exhibited higher all-cause mortality risks 82% increased risk (95% CI [65, 101]; *p* < 0.001)compared to never-smokers [23]. The increased risk of low-intensity and other outcomes other than cardiovascular outcomes, such as cancer, has also been reported [24].

Comparing the associations within pack-year categories between former and current smokers, it was observed that the magnitude of health risk for former smokers within the highest pack-year group (>20 pack-years) was lower than the magnitude of health risk for current smokers within the lowest pack-year group (≤5 pack-years). This finding reinforces the argument that pack-years alone may not sufficiently capture the complexities of smoking-related cardiovascular risk [25]. Incorporating both smoking status and pack-years as combined measures of smoking intensity offers a more nuanced understanding of the relationship between smoking behaviors and cardiovascular outcomes. This approach acknowledges the significant differences in risk between current and former smokers and underscores the importance of considering both the quantity and duration of smoking in risk assessments. Notably, the dose-response curve for smoking intensity and its associated health outcomes did not plateau until reaching the thresholds of approximately 20 CPD and 20 pack-years.

Quitting smoking is a critical step for individuals who smoke to lower their cardiovascular risk [25]. Although evidence varies regarding the timeline for excess cardiovascular risk reduction following smoking cessation, most studies suggest a return to levels comparable to never-smokers after 20 years of cessation [15,17,18]. Our study findings showed that former smokers exhibited risk reductions of greater than 80% compared to current smokers within two decades of smoking cessation. Moreover, it is important to note that the cubic spline analysis showed that the reduction in risk starts immediately after quitting and continues to decrease substantially up to 20 years post-cessation, and every attempt should be taken into account to encourage early quitting. The concurrent evaluation of cessation duration and smoking pack-years indicates that the time elapsed since quitting holds greater importance for risk estimation than the total pack-years. Moreover, quitting five years earlier may compensate for a significant portion of the risk associated with an extensive smoking history. Moreover, our results showed that the effect of increasing pack-years is more prominent in mortality outcomes than cardiovascular outcomes, which emphasizes the strong cumulative effect of smoking on total mortality [26].

Comparison of the two eras shows an increase of the smoking–cardiovascular dose–response relationship over time. The observed temporal trend of increasing smoking-related cardiovascular risks represents a concerning epidemiological phenomenon that warrants careful consideration of multiple contributing factors. The heightened HRs among participants enrolled after 2001 may reflect several interconnected mechanisms, including evolving smoking behaviors and patterns and improvements in baseline cardiovascular health among never-smokers that accentuate the relative harm of smoking exposure. This finding is consistent with multiple papers by leaders in tobacco epidemiology that have demonstrated relative risks of all-cause and cardiovascular disease mortality have increased over time, as documented in the British Doctors Study [27] and analyses of 50-year smoking trends in the United States [17]. These temporal increases in relative risk have been largely attributed to very rapid falls in mortality rates among never smokers and less rapid falls (or even stagnation) in rates among people who smoke. This phenomenon has been linked to the “maturing” of the tobacco epidemic, whereby people who smoke in the most recent birth cohorts have commenced smoking at younger ages and have smoked heavily throughout their lives, whereas people who smoke in earlier cohorts, on average, started smoking at later ages and smoked less on average. The temporal intensification of smoking’s cardiovascular impact suggests that contemporary smokers may face even greater health risks compared to their never-smoking peers than previously estimated from earlier cohort studies, underscoring the critical importance of smoking cessation interventions and reinforcing the public health imperative to prevent smoking initiation in younger populations who may be exposed to these heightened risk profiles throughout their lifetime. Our study has several strengths. First, CCC-Tobacco is an integration of highly detailed, primarily U.S.-based cohorts focusing on cardiovascular health and aging. Additionally, the prolonged observation period of up to 74 years significantly improves our capability to detect even small associations. Moreover, this comprehensive and large study population provided a precise effect size estimate of low-burden smoking and precise shapes of associations that could be utilized as robust evidence for regulatory authorities in making informed decisions. Secondly, by harmonizing various cohorts with respect to specific demographic criteria, as well as racial and ethnic categories, we achieved a representation in which women constituted the majority of the study population. Additionally, we included diverse racial and ethnic groups, including White, Black or African American, Hispanic or Latino, and American Indian or Alaskan Native. Third, to the best of our knowledge, this is the first study to investigate the concurrent influence of cessation duration and smoking pack-years on different outcomes. It is also important to notice that the CCC-Tobacco working group will continue to extend this work with a special focus on age, sex, and race disparities regarding the associations of smoking burden, smoking intensity, and time since quitting with the various cardiovascular and mortality outcomes.

This study has limitations. First, smoking status was determined based on self-reported data from individual cohort surveys. The stigma attached to smoking could lead to underreporting of current smoking status, particularly among women, affecting the accuracy of the data. Nevertheless, it is important to acknowledge that self-reported measures of smoking behavior are the current standard in clinical practice settings [28,29]. Moreover, data regarding other tobacco products and electronic nicotine delivery systems were not available for all the participants of the study population; hence, we could not investigate other use patterns, such as dual- and poly-use, in our analysis. Given the observational study design, we cannot exclude residual confounding, including but not limited to types of cigarettes and time cohort effects.

Our study spans over 50 years, and since smoking-related risks have changed over successive decades, our estimates represent an average across time and may underestimate or overestimate current risks. However, we have conducted a sensitivity analysis based on the baseline enrollment date of the participant, stratifying them into before and after enrollment in 2001 to clarify the distinction of the relative risk in these two different periods, although this analysis should also be interpreted with caution, as different decades are represented by different cohorts, introducing some inherent variability. Additionally, we analyzed data spanning multiple years of follow-up but could not fully explore how the relationship between the use of tobacco products and cardiovascular disease outcomes has evolved over time, especially considering the evolution of the tobacco industry and tobacco regulation.

Due to variability in baseline cardiovascular disease ascertainment across cohorts, we could not consistently exclude all participants with all prevalent cardiovascular conditions (e.g., CHD, stroke, AFib, HF) from the primary analysis. Therefore, our primary estimates could reflect a mixture of incidence and recurrent cardiovascular events. Although we addressed this issue through adjustment and sensitivity analyses, excluding individuals with known prevalent disease at baseline, residual bias from incomplete exclusions might persist. Comparison between Tables 4 and S5, however, shows that there are comparable risk estimates generated from each approach. However, emerging evidence has demonstrated that most predictors, including smoking status, show similar associations with MACE regardless of baseline atherosclerotic CVD status and have emphasized universal risk prediction models [30].

We acknowledge limitations in estimating the relationship between time since quitting and health outcomes, particularly at older ages, where the “sick quitter effect” becomes more pronounced. While our sensitivity analyses provide reassurance regarding the overall robustness of our findings, caution is warranted when interpreting the precise temporal relationship between smoking cessation and risk reduction over extended timeframes. This study’s analysis of “time since quitting” smoking is limited by the challenge of time-varying duration. Younger individuals rarely exhibit long quit durations, violating the positivity assumption and confounding results with age-related biases. Additionally, left-censoring due to mortality skews the distribution, as older individuals with shorter quit times are underrepresented, creating potential selection bias. Moreover, due to the unavailability of data on other causes of mortality, including cancer mortality, a competing risk analysis was not conducted. Lastly, our reliance on a single baseline self-reported measurement of smoking exposure, combined with the substantial time gap between this assessment and outcome measurement, introduces potential exposure misclassification as smoking behaviors likely changed for many participants during follow-up. This regression dilution bias may have attenuated the observed associations. Prior cohort studies suggest that using a single measurement of smoking can underestimate true relative risks by up to 15%, particularly for intensity-related exposures [17,31]. Future work in CCC-Tobacco will seek to harmonize tobacco use data across all visits for all cohorts, which will enable the study of product transitions, including cessation. We reported comprehensive smoking behavior measures and their association with various cardiovascular, cause-specific, and all-cause mortality outcomes. The results of our study showed that although former smokers with cumulative exposure below 5 pack-years did not show statistically significant associations, continuous dose-response analyses and findings among current low-intensity smokers underscore that no threshold of exposure is risk-free. Our results indicate that there is a substantial risk reduction within the first 20 years of smoking cessation. However, even after 21–30 years of cessation, former smokers may still exhibit higher risks compared to those who never smoked. These findings suggest that health authorities should emphasize both smoking cessation and the prevention of smoking initiation. Our findings reinforce well‐established public health guidance—that recent cessation drives substantially greater cardiovascular and mortality risk reductions than intensity reduction alone. Consistent with WHO recommendations, complete quitting should remain the principal goal, with any reductions in smoking intensity viewed only as interim steps toward full cessation. The epidemiological findings from this study can aid in shaping regulatory strategies and intervention guidelines against smoking. Additionally, they provide a framework for future research to delve into the complex dynamics between smoking habits, various population subgroups, and their associations with cardiovascular health outcomes. As part of the CCC-Tobacco project, future projects will focus on age, sex, and race disparities the evaluated association of smoking parameters, and a comprehensive set of cardiovascular and mortality outcomes.

## Supporting information

S1 Table Characteristics of the 23 participating cohorts of the cross-cohort collaboration-tobacco dataset. (DOCX)

S2 Table Baseline characteristics of the included cohorts (parts 1&2). (DOCX)

S3 Table Characteristics of smoking parameters by cohorts. (DOCX)

S4 Table Outcomes follow-up time in each cohort. (DOCX)

S5 Table Association between smoking status and incidence of cardiovascular and mortality outcomes, excluding patients reported baseline cardiovascular disease. (DOCX)

S6 Table Association between smoking status and incidence of cardiovascular and mortality outcomes in participants older than 60 years old. (DOCX)

S7 Table Association between smoking status and incidence of cardiovascular and mortality outcomes in participants aged younger or equal to 60 years old. (DOCX)

S8 Table Association between pack years with cardiovascular outcomes among former cigarette users. (DOCX)

S9 Table Association between pack years with cardiovascular outcomes among current cigarette users. (DOCX)

S10 Table Association between the number of cigarettes per day with cardiovascular outcomes among current cigarette users. (DOCX)

S1 Fig CVD and mortality outcomes hazard ratios (HRs) by years since quit smoking among former smokers compared with current and never smokers, excluding baseline CHD and considering recent quitters (within 2 years) as current smokers. MI, myocardial infarction; CHD, coronary heart disease; CVD, cardiovascular disease; HF, heart failure, AFib, atrial fibrillation. Current smokers presented with a red dot-dashed line. The reference group is never-smokers presented with a dotted black line.
(DOCX)

S2 Fig CVD and mortality outcomes hazard ratios (HRs) by years since quit smoking among former smokers compared with current and never smokers among participants equal to or younger than 60 years old. MI, myocardial infarction; CHD, coronary heart disease; CVD, cardiovascular disease; HF, heart failure, AFib, atrial fibrillation. Current smokers presented with a red dot-dashed line. The reference group is never-smokers presented with a dotted black line.
(DOCX)

S3 Fig CVD and mortality outcomes hazard ratios (HRs) by years since quit smoking among former smokers compared with current and never smokers among participants older than 60 years old. MI, myocardial infarction; CHD, coronary heart disease; CVD, cardiovascular disease; HF, heart failure, AFib, atrial fibrillation. Current smokers presented with a red dot-dashed line. The reference group is never-smokers presented with a dotted black line.
(DOCX)

S1 STROBE Checklist STROBE checklist. Adapted from the STrengthening the Reporting of OBservational studies in Epidemiology (STROBE) Statement checklist, available at https://www.strobe-statement.org/. Licensed under Creative Commons Attribution 4.0 International License (CC BY 4.0).
(DOCX)

## Abbreviations

AFib: atrial fibrillation
BMI: body mass index
CCC: Cross-Cohort Collaboration
CHD: coronary heart disease
CI: confidence interval
CPD: cigarettes per day
CVD: cardiovascular disease
HF: heart failure
HR: hazard ratio
MI: myocardial infarction
