# Supplementary material for: Association between cigarette smoking status, intensity, and cessation duration with long-term incidence of nine cardiovascular and mortality outcomes: The Cross-Cohort Collaboration (CCC)
Source: PLoS Med. 2025 Nov 18;22(11):e1004561. doi: 10.1371/journal.pmed.1004561 (PMC12626310; doi:10.1371/journal.pmed.1004561)
Supplement: S1 Table — (DOCX) [file pmed.1004561.s001.docx]

| S1 Table. Characteristics of the Twenty-three Participating Cohorts of the Cross-Cohort Collaboration-Tobacco Dataset | | | |
| --- | --- | --- | --- |
| Participating cohort (website link) | **Cohort description** | **Enrollment years** |  |
| Traditional cardiovascular cohorts | | |  |
| Atherosclerosis Risk in Communities Study ([ARIC](https://aric.cscc.unc.edu/aric9/)) | 15792 in 4 US communities aged 45-64 | 1987 |  |
| Coronary Artery Risk Development in Young Adults ([CARDIA](https://www.cardia.dopm.uab.edu/)) | 5115 at 4 US field centers aged 18-30 years | 1985-86 |  |
| Cardiovascular Health Study ([CHS](https://chs-nhlbi.org/)) | 5888 adults aged 65 or older in 4 US communities | 1989-1999 |  |
| Dallas Heart Study ([DHS](https://www.utsouthwestern.edu/education/medical-school/departments/internal-medicine/research/dallas-heart/)) | 3,557 persons of from multiethnic cohort supported by the Hoffman Family Center, and the National Center for Advancing Translational Sciences (NCATS) with annual surveys within hospitals in the DFW metroplex. | 2000 |  |
| Framingham Heart Study ([FHSL](https://www.framinghamheartstudy.org/)) | 5209 adult population of Framingham Massachusetts aged 30-62 (Original cohort) | 1948 |  |
|  | Offspring cohort: 5124 adult children of the original cohort and their spouses aged 30-74 | 1971 |  |
|  | FHS 3rd Gen: 4095 men and women aged >19 years with ≥one parent in the offspring study | 2002 |  |
| Jackson Heart Study ([JHS](https://www.jacksonheartstudy.org/)) | 5306 community-based African Americans from 3 counties in Jackson MS aged 35-84 | 2000-2004 |  |
| Multi-Ethnic Study of Atherosclerosis ([MESA](https://www.mesa-nhlbi.org/)) | More than 6000 multi-ethnic men and women from 6 communities in the US aged 45-84 | 2000 to 2002 |  |
| The Multiple Risk Factor Intervention Trial ([MRFIT](https://biolincc.nhlbi.nih.gov/studies/mrfit/)) | 12866 men aged 35-57 enrolled in coronary heart disease intervention trial | 1972 |  |
| Reasons for Geographic and Racial Differences in Stroke ([REGARDS](https://www.uab.edu/soph/regardsstudy/)) | 30239 employed men and women ≥ 45 years | 2003 |  |
| Strong Heart Study ([SHS](https://strongheartstudy.org/)) | 4500 American Indian tribal members aged 35-74 years | 1988 |  |
| Non-cardiovascular specific cohorts | | |  |
| Baltimore Longitudinal Study of Aging ([BLSA](https://www.blsa.nih.gov/)) | >3000 men and women > 20 years | 1958 |  |
| Chronic Renal Insufficiency Cohort Study ([CRIC](http://www.cristudy.org/Chronic-Kidney-Disease/Chronic-Renal-Insufficiency-Cohort-Study/)) | 3939 with chronic kidney disease (1560 older adults during third phase) | 2001-13 (I & II)  2013-15 (III) |  |
| Brazilian Longitudinal Study of Adult Health ([ELSA-Brasil](https://pubmed.ncbi.nlm.nih.gov/22234482/)) | 15000 active & retired civil servants from teaching & research institutions aged 35-74 years | 2008 |  |
| Health Aging and Body Composition Study ([Health ABC](https://healthabc.nia.nih.gov/)) | 3075 community-dwelling in Memphis TN or Pittsburgh PA and aged 70–79 years | 1997 |  |
| The Osteoporotic Fractures in Men Study ([MrOS](https://mrosonline.ucsf.edu/)) | 6000 senior men 65 years and older from 6 US communities | 2000 |  |
| Rancho Bernardo Study ([RBS](https://knit.ucsd.edu/ranchobernardostudy/)) of Healthy Aging | 6339 Community based cohort of all residents of Rancho Bernardo | 1972-1974 |  |
| The Study of Osteoporotic Fractures ([SOF](https://sofonline.ucsf.edu/)) | 10366 older women (65 or older) | 1986 |  |
| Study of Women’s Health Across the Nation ([SWAN](https://www.swanstudy.org/)) | 3302 women in longitudinal study of women’s health in 7 US research centers | 1996-1997 |  |
| Women’s Health Initiative ([WHI](https://www.whi.org/)) | 161808 postmenopausal women aged 50-79 years | 1993 |  |
