# Supplementary material for: Association between cigarette smoking status, intensity, and cessation duration with long-term incidence of nine cardiovascular and mortality outcomes: The Cross-Cohort Collaboration (CCC)
Source: PLoS Med. 2025 Nov 18;22(11):e1004561. doi: 10.1371/journal.pmed.1004561 (PMC12626310; doi:10.1371/journal.pmed.1004561)
Supplement: S2 Table — (DOCX) [file pmed.1004561.s002.docx]

**S2A Table. Baseline characteristics of the included cohorts**

| **Variable** | **ARIC** | **BLSA** | **CARDIA** | **CRIC** | **CHS original** | **CHS supplemental** | **DHS** | **ELSA-Brazil** | **FHS_original** | **FHS_offspring** | **FHS_Gen3** |
| --- | --- | --- | --- | --- | --- | --- | --- | --- | --- | --- | --- |
| **Sample Size, N(%)** | 15792 (4.82) | 1793 (0.55) | 4352 (1.33) | 5625 (1.72) | 5201 (1.59) | 687 (0.21) | 3557 (1.09) | 15105 (4.61) | 4068 (1.24) | 4856 (1.48) | 4064 (1.24) |
| **Age, mean (SD)** | 54.16 (5.76) | 65.71 (15.17) | 29.97 (3.64) | 59.57 (10.74) | 72.77 (5.61) | 72.99 (5.79) | 43.92 (10.11) | 52.09 (9.08) | 55.55 (8.44) | 36.85 (9.90) | 40.16 (8.83) |
| **Female, N(%)** | 8710 (55.2) | 926 (51.6) | 2393 (55.0) | 2457 (43.7) | 2962 (57.0) | 431 (62.7) | 1986 (55.8) | 8218 (54.4) | 2296 (56.4) | 2509 (51.7) | 2168 (53.3) |
| **Race and Ethnicity*** |  |  |  |  |  |  |  |  |  |  |  |
| **White** | 11478 (72.7) | 1272 (74.3) | 2233 (51.3) | 2276 (41.9) | 4925 (94.9) | 0 (0.0) | 1047 (29.4) | 7791 (52.2) | 4068 (100.0) | 4856 (100.0) | 4064 (100.0) |
| **Black or African American** | 4266 (27.0) | 406 (23.7) | 2119 (48.7) | 2422 (44.6) | 246 (4.7) | 678 (99.9) | 1834 (51.6) | 6599 (44.2) | 0 (0.0) | 0 (0.0) | 0 (0.0) |
| **Education** |  |  |  |  |  |  |  |  |  |  |  |
| **High School not completed** | 3767 (23.9) | 18 (1.0) | 197 (4.5) | 1142 (20.3) | 1438 (27.7) | 294 (43.0) | 726 (20.4) | 1922 (12.7) | 1620 (41.0) | 304 (8.9) | 22 (0.7) |
| **High school completed** | 6412 (40.7) | 149 (8.4) | 2160 (49.8) | 1035 (18.4) | 1432 (27.6) | 151 (22.1) | 1106 (31.1) | 5233 (34.6) | 1204 (30.5) | 1719 (50.3) | 465 (13.7) |
| **College degree** | 5586 (35.4) | 1615 (90.6) | 1983 (45.7) | 3446 (61.3) | 2317 (44.7) | 239 (34.9) | 1724 (48.5) | 7950 (52.6) | 1123 (28.5) | 1396 (40.8) | 2897 (85.6) |
| **BMI, kg/m2** | 27.71 (5.37) | 27.03 (4.92) | 26.15 (5.91) | 32.30 (7.64) | 26.41 (4.52) | 28.78 (5.65) | 29.60 (7.04) | 27.02 (4.75) | 25.87 (4.14) | 25.20 (4.31) | 26.91 (5.57) |
| **Alcohol use*, N (%)** | 8768 (55.8) | 1474 (82.5) | 3606 (83.3) | 3468 (61.7) | 2697 (52.1) | 230 (33.8) | 2434 (68.6) | 7245 (48.0) | 2892 (71.4) | 4166 (86.6) | 3367 (82.9) |
| **Hypertension, N (%)** | 5511 (34.9) | 695 (39.6) | 197 (4.5) | 5228 (92.9) | 3336 (64.2) | 554 (80.6) | 1166 (32.8) | 5585 (37.0) | 1974 (48.6) | 953 (19.7) | 673 (16.6) |
| **BP medication* N (%)** | 4003 (25.5) | 635 (35.7) | 70 (1.6) | 5177 (92.2) | 2348 (45.2) | 442 (64.5) | 767 (22.1) | 4412 (29.2) | 377 (9.3) | 160 (3.3) | 343 (8.5) |
| **Diabetes N (%)** | 1561 (10.0) | 269 (15.0) | 83 (1.9) | 2850 (51.3) | 763 (14.8) | 162 (24.9) | 374 (10.6) | 3010 (19.9) | 171 (4.2) | 91 (2.0) | 123 (3.0) |
| **Dyslipidemia N (%)** | 8986 (57.8) | 841 (50.8) | 1379 (32.5) | 4370 (86.4) | 2674 (51.7) | 281 (42.6) | 1846 (52.0) | 8158 (54.0) | 2355 (60.1) | 2221 (46.5) | 1557 (38.3) |
| **Hyperlipidemia N (%)** | 5771 (37.1) | 233 (14.8) | 411 (9.7) | 1090 (27.8) | 1772 (34.3) | 175 (26.6) | 654 (18.4) | 5234 (34.7) | 2346 (59.9) | 1135 (23.8) | 671 (16.5) |
| **Lipid Lowering Medication N (%)** | 448 (2.9) | 529 (58.8) | 11 (0.3) | 3454 (61.9) | 97 (1.9) | 35 (5.1) | 238 (6.9) | 1978 (13.1) | 46 (1.1) | 27 (0.6) | 273 (6.7) |
| **Systolic BP, mmHg** | 121.35 (18.97) | 118.26 (15.90) | 107.82 (11.58) | 128.56 (21.42) | 135.78 (21.53) | 143.82 (23.21) | 124.96 (18.92) | 123.61 (16.70) | 137.99 (22.77) | 122.10 (16.49) | 116.77 (14.12) |
| **Diastolic BP, mmHg** | 73.75 (11.33) | 66.58 (8.85) | 69.25 (10.15) | 71.12 (12.58) | 70.10 (11.53) | 76.57 (11.81) | 78.34 (10.26) | 74.49 (9.84) | 85.10 (11.56) | 78.76 (10.87) | 75.31 (9.66) |
| **Total Cholesterol, mg/dL** | 214.95 (42.07) | 189.93 (36.46) | 178.08 (34.34) | 183.72 (45.58) | 211.37 (39.25) | 209.86 (39.44) | 180.32 (39.62) | 214.73 (42.66) | 252.89 (48.25) | 200.21 (40.01) | 188.82 (35.50) |
| **LDL-C4, mg/dL** | 137.64 (39.35) | 109.70 (32.29) | 108.46 (32.06) | 102.74 (35.58) | 129.82 (35.64) | 129.84 (36.11) | 106.29 (35.40) | 131.00 (35.12) | NaN (NA) | 128.60 (37.22) | 111.74 (31.45) |
| **HDL-C, mg/dL** | 51.59 (17.10) | 59.65 (17.05) | 53.27 (14.15) | 47.50 (15.48) | 53.82 (15.74) | 57.23 (15.46) | 49.84 (14.79) | 56.70 (14.57) | NaN (NA) | 51.85 (16.24) | 54.33 (16.09) |

**S2B Table. Baseline characteristics of the included cohorts**

| **Variable** | "HCHS SOl" | HEALTHABC | JHS | MESA | MRFIT | MROS | Rancho Bernardo | REGARDS | SHS | SOF | SWAN | WHI clinical trial | WHI Observational |
| --- | --- | --- | --- | --- | --- | --- | --- | --- | --- | --- | --- | --- | --- |
| **Sample Size, N(%)** | 16415 (5.01) | 3075 (0.94) | 5306 (1.62) | 6814 (2.08) | 12866 (3.93) | 5994 (1.83) | 2480 (0.76) | 30183 (9.22) | 3501 (1.07) | 9704 (2.96) | 3302 (1.01) | 68132 (20.80) | 93676 (28.60) |
| **Age, mean (SD)** | 45.85 (13.93) | 73.63 (2.87) | 54.85 (12.86) | 62.15 (10.23) | 46.18 (5.96) | 73.66 (5.87) | 70.07 (11.07) | 64.85 (9.43) | 56.45 (8.14) | 71.62 (5.22) | 45.85 (2.69) | 62.71 (7.01) | 63.62 (7.37) |
| **Female, N(%)** | 9835 (59.9) | 1584 (51.5) | 3367 (63.5) | 3601 (52.8) | 0 (0.0) | 0 (0.0) | 1386 (55.9) | 16632 (55.1) | 2049 (58.5) | 9704 (100.0) | 3302 (100.0) | 68132 (100.0) | 93676 (100.0) |
| **Race and Ethnicity** |  |  |  |  |  |  |  |  |  |  |  |  |  |
| **White** | 0 (0.0) | 1794 (58.3) | 0 (0.0) | 2622 (38.5) | 11559 (89.8) | 5385 (89.8) | 2453 (100.0) | 17669 (58.5) | 0 (0.0) | 9671 (100.0) | 1551 (47.0) | 57528 (84.4) | 80100 (85.5) |
| **Black or African American** | 0 (0.0) | 1281 (41.7) | 5306 (100.0) | 1893 (27.8) | 931 (7.2) | 244 (4.1) | 0 (0.0) | 12514 (41.5) | 0 (0.0) | 0 (0.0) | 934 (28.3) | 6826 (10.0) | 7501 (8.0) |
| **American Indian or Alaskan Native** | 0 (0.0) | 0 (0.0) | 0 (0.0) | 0 (0.0) | 0 (0.0) | 0 (0.0) | 0 (0.0) | 0 (0.0) | 3501 (100) | 0 (0.0) | 0 (0.0) | 0 (0.0) | 0 (0.0) |
| **Education** |  |  |  |  |  |  |  |  |  |  |  |  |  |
| **High School not completed** | 6207 (38.0) | 775 (25.3) | 973 (18.4) | 1225 (18.0) | 2083 (16.3) | 393 (6.6) | 149 (6.1) | 3792 (12.6) | 1472 (42.1) | 2214 (22.9) | 238 (7.3) | 3795 (5.6) | 4849 (5.2) |
| **High school completed** | 4180 (25.6) | 1000 (32.6) | 1065 (20.1) | 1236 (18.2) | 2685 (21.0) | 1036 (17.3) | 613 (25.1) | 7804 (25.9) | 985 (28.2) | 3807 (39.3) | 581 (17.8) | 12502 (18.5) | 15122 (16.3) |
| **College degree** | 5937 (36.4) | 1292 (42.1) | 3248 (61.4) | 4330 (63.8) | 8035 (62.8) | 4565 (76.2) | 1676 (68.7) | 18562 (61.5) | 1039 (29.7) | 3654 (37.8) | 2452 (75.0) | 51386 (75.9) | 72938 (78.5) |
| **BMI, kg/m2** | 29.78 (6.07) | 27.39 (4.82) | 31.75 (7.24) | 28.34 (5.48) | 27.71 (3.46) | 27.38 (3.83) | 24.85 (3.67) | 29.32 (6.21) | 30.47 (6.04) | 26.40 (4.47) | 28.26 (7.21) | 28.94 (5.90) | 27.27 (5.87) |
| **Alcohol use*, N (%)** | 7750 (47.4) | 1526 (49.7) | 2419 (45.8) | 3749 (55.4) | 11897 (92.5) | 3865 (64.6) | 2190 (90.5) | 11043 (37.3) | 1392 (90.6) | 6788 (70.0) | 1345 (47.4) | 24393 (36.1) | 35528 (38.2) |
| **Hypertension, N (%)** | 4476 (27.3) | 2116 (68.8) | 3135 (59.2) | 3301 (48.5) | 11788 (91.6) | 4206 (70.9) | 1473 (59.5) | 17854 (59.2) | 1317 (37.8) | 6066 (62.5) | 790 (24.0) | 22831 (33.5) | 28611 (30.6) |
| **BP medication* N (%)** | 2654 (16.6) | 1672 (54.6) | 2754 (52.4) | 2536 (37.2) | 2488 (19.3) | 3054 (51.0) | 777 (37.6) | 15551 (53.6) | 388 (18.3) | 2652 (30.3) | 471 (14.3) | 9575 (14.1) | 10172 (10.9) |
| **Diabetes N (%)** | 3285 (20.2) | 1015 (33.0) | 1242 (23.7) | 859 (12.7) | 711 (5.6) | 881 (15.8) | 257 (10.4) | 6398 (22.0) | 1442 (41.5) | 682 (7.0) | 127 (4.1) | 4300 (6.3) | 5318 (5.7) |
| **Dyslipidemia N (%)** | 9798 (60.3) | 1616 (53.1) | 2828 (57.7) | 3787 (55.7) | 10391 (80.8) | 3310 (58.5) | 1074 (43.6) | 19166 (65.4) | 2186 (63.5) | 554 (73.2) | 1425 (43.5) | 9450 (84.2) | 9273 (94.6) |
| **Hyperlipidemia N (%)** | 4524 (27.8) | 850 (28.0) | 1124 (23.3) | 1559 (23.0) | 8970 (69.7) | 1475 (26.7) | 911 (37.0) | 6825 (23.6) | 906 (26.3) | 450 (59.4) | 580 (17.7) | 9113 (14.9) | 13998 (15.3) |
| **Lipid Lowering Medication N (%)** | 1990 (12.4) | 437 (14.3) | 721 (13.7) | 1100 (16.2) | 159 (1.2) | 1541 (25.7) | 16 (0.8) | 10010 (33.5) | 12 (0.6) | 0 (NaN) | 34 (1.0) | 7223 (10.6) | 8835 (9.4) |
| **Systolic BP, mmHg** | 121.79 (18.06) | 135.86 (21.04) | 127.48 (16.90) | 126.59 (21.48) | 147.58 (15.36) | 139.21 (18.71) | 138.77 (22.08) | 127.60 (16.69) | 126.66 (19.14) | 141.81 (19.21) | 117.85 (17.03) | 127.97 (17.40) | 126.96 (17.98) |
| **Diastolic BP, mmHg** | 72.98 (11.01) | 71.42 (11.74) | 75.70 (8.78) | 71.91 (10.26) | 99.20 (7.68) | NaN (NA) | 76.23 (9.54) | 76.55 (9.73) | 76.45 (10.21) | 76.76 (9.24) | 75.48 (10.52) | 75.88 (9.12) | 74.74 (9.34) |
| **Total Cholesterol, mg/dL** | 199.22 (44.14) | 202.81 (38.58) | 199.31 (40.10) | 194.16 (35.73) | 240.44 (36.80) | 193.27 (34.23) | 219.34 (40.45) | 192.10 (40.15) | 195.46 (39.56) | 239.08 (40.12) | 194.57 (34.89) | 223.70 (38.78) | 218.19 (36.96) |
| **LDL-C4, mg/dL** | 122.62 (36.63) | 121.54 (34.65) | 126.65 (36.61) | 117.20 (31.46) | 160.02 (36.03) | 114.17 (30.99) | 134.50 (36.86) | 113.93 (34.80) | 110.42 (32.00) | 152.02 (36.09) | 116.11 (31.01) | 135.97 (35.78) | 125.37 (34.34) |
| **HDL-C, mg/dL** | 49.19 (13.02) | 54.09 (17.02) | 51.77 (14.64) | 50.96 (14.83) | 42.09 (11.78) | 48.90 (14.62) | 61.71 (18.81) | 51.79 (16.17) | 46.20 (13.91) | 53.14 (14.86) | 55.90 (14.56) | 57.91 (15.06) | 63.01 (16.96) |
| BMI: body mass index; BP: blood pressure; HPL: hyperlipidemia; HTG: hypertriglyceridemia; LDL-C: low density lipoprotein cholesterol; HDL-C: high density lipoprotein cholesterol  *other race/ethnicities were not included on this table since some cohorts has below 10 individuals.  ARIC: Atherosclerosis Risk in Communities Study, CARDIA: Coronary Artery Risk Development in Young Adults, CHS: Cardiovascular Health Study, DHS: Dallas Heart Study, FHSL: Framingham Heart Study, JHS: Jackson Heart Study, MESA: Multi-Ethnic Study of Atherosclerosis, MRFIT: The Multiple Risk Factor Intervention Trial, REGARDS: Reasons for Geographic and Racial Differences in Stroke, SHS: Strong Heart Study, BLSA: Baltimore Longitudinal Study of Aging, CRIC: Chronic Renal Insufficiency Cohort Study, ELSA-Brasil: Brazilian Longitudinal Study of Adult Health, Health ABC: Health Aging and Body Composition Study, MrOS: The Osteoporotic Fractures in Men Study, RBS: Rancho Bernardo Study of Healthy Aging, SOF: The Study of Osteoporotic Fractures, SWAN: Study of Women's Health Across the Nation, WHI: Women's Health Initiative. | | | | | | | | | | | | | |
