# Supplementary material for: Association between cigarette smoking status, intensity, and cessation duration with long-term incidence of nine cardiovascular and mortality outcomes: The Cross-Cohort Collaboration (CCC)
Source: PLoS Med. 2025 Nov 18;22(11):e1004561. doi: 10.1371/journal.pmed.1004561 (PMC12626310; doi:10.1371/journal.pmed.1004561)
Supplement: S3 Table — (DOCX) [file pmed.1004561.s003.docx]

**S3 Table.** **Characteristics of smoking parameters by cohorts.**

| **Cohort** | **Average smoking intensity among current users (SD)** | **Average pack-years among ever users (SD)** | **Average cessation time (SD)** |
| --- | --- | --- | --- |
| **ARIC** | 20.65 (12.33) | 36.21 (24.18) | 14.42 (10.51) |
| **BLSA** | 12.54 (6.74) | 18.83 (20.43) | 30.17 (13.66) |
| **CHS** | 16.97 (9.99) | 34.47 (28.51) | 21.05 (13.73) |
| **CARDIA** | 13.07 (9.02) | 8.51 (8.39) | 6.11 (4.79) |
| **CRIC** | 8.41 (7.05) | 20.92 (22.34) | 19.56 (13.55) |
| **DHS** | 12.41 (15.43) | 15.97 (22.10) | 14.47 (10.83) |
| **ELSA-Brasil** | 13.21 (9.70) | 19.92 (20.63) | 17.09 (11.06) |
| **FHS-Original** | 20.21 (12.31) | 21.18 (16.39) | 19.96 (9.79) |
| **FHS-Offspring** | 21.46 (12.52) | 20.08 (18.50) | 7.95 (6.03) |
| **FHS-Gen III** | 14.34 (9.64) | 17.34 (15.57) | 13.88 (9.21) |
| **GOLDN** | 13.17 (9.57) | 19.03 (20.60) | 22.01 (14.07) |
| **JHS** | 12.79 (9.05) | 21.20 (20.07) | 16.38 (11.19) |
| **Health ABC** | 16.70 (10.55) | 34.22 (30.07) | 25.05 (14.30) |
| **MESA** | 14.02 (16.88) | 23.85 (27.19) | 22.42 (13.19) |
| **MrOS** | 21.86 (9.95) | 30.46 (26.31) | 29.10 (14.14) |
| **REGARDS** | 22.42 (20.30) | 32.18 (36.58) | 23.84 (13.65) |
| **RBS** | 19.41 (12.12) | 30.76 (28.10) | NA |
| **SHS** | 13.19 (12.68) | 24.86 (39.10) | 16.25 (12.14) |
| **SWAN** | 15.09 (8.85) | 15.67 (13.43) | 13.56 (8.10) |
| **SOF** | 16.40 (7.62) | 27.15 (24.16) | 20.18 (13.10) |
| **WHI-Clinical Trial** | 15.06 (10.20) | 22.04 (22.41) | 21.39 (12.79) |
| **WHI-Observation Study** | 14.60 (10.30) | 21.65 (22.43) | 22.08 (12.78) |
| ARIC: Atherosclerosis Risk in Communities Study, CARDIA: Coronary Artery Risk Development in Young Adults, CHS: Cardiovascular Health Study, DHS: Dallas Heart Study, FHSL: Framingham Heart Study, JHS: Jackson Heart Study, MESA: Multi-Ethnic Study of Atherosclerosis, MRFIT: The Multiple Risk Factor Intervention Trial, REGARDS: Reasons for Geographic and Racial Differences in Stroke, SHS: Strong Heart Study, BLSA: Baltimore Longitudinal Study of Aging, CRIC: Chronic Renal Insufficiency Cohort Study, ELSA-Brasil: Brazilian Longitudinal Study of Adult Health, Health ABC: Health Aging and Body Composition Study, MrOS: The Osteoporotic Fractures in Men Study, RBS: Rancho Bernardo Study of Healthy Aging, SOF: The Study of Osteoporotic Fractures, SWAN: Study of Women's Health Across the Nation, WHI: Women's Health Initiative. | | | |
