# Supplementary material for: Association between cigarette smoking status, intensity, and cessation duration with long-term incidence of nine cardiovascular and mortality outcomes: The Cross-Cohort Collaboration (CCC)
Source: PLoS Med. 2025 Nov 18;22(11):e1004561. doi: 10.1371/journal.pmed.1004561 (PMC12626310; doi:10.1371/journal.pmed.1004561)
Supplement: S4 Table — (DOCX) [file pmed.1004561.s004.docx]

| **S4 Table.** Outcomes follow-up time in each cohort. | | | | | | | | | | | | |
| --- | --- | --- | --- | --- | --- | --- | --- | --- | --- | --- | --- | --- |
|  |  |  | Follow Up Time in years | | | | | | | | | |
| Cohorts | **Baseline visit for the CCC- Tobacco** | **The baseline date for the Cox models** | **MI** | **Stroke** | **Heart failure** | **Atrial fibrillation** | **CVD** | **CHD** | **CHD mortality** | **CVD mortality** | **All-cause mortality** |  |
| [ARIC](https://www5.cscc.unc.edu/aric9/sites/default/files/public/visitdocuments/v10/Manual%201%20General%20Description%20230508.pdf) | Visit 1 | 1987-1989 | 27 (17 - 31) | 26 (16 - 30) | 25 (15 - 30) | 26 (16 - 30) | 25 (16 - 29) | 28 (18 - 31) | 28 (18 - 31) | 28 (18 - 31) | 26 (16 - 30) |  |
| BLSA | Visit 1 | 1958 | 9 (5 - 12) | 9 (5 - 12) | 9 (5 - 12) | NA | NA | NA | NA | 10 (6 - 13) | 9 (5 - 12) |  |
| CARDIA | Year 5 | 1990-1991 | 27 (27 - 27) | 27 (27 - 27) | 27 (27 - 27) | 27 (27 - 27) | 25 (20 - 25) | 27 (27 - 27) | 27 (27 - 27) | 27 (27 - 27) | 27 (27 - 27) |  |
| CHS original | Visit 2 | 1991-1992 | 12 (7-18) | 12 (7-18) | 11 (6-18) | 11 (6-17) | 11 (6-17) | 12 (6-18) | 13 (7-19) | 13 (7-19) | 13 (7-19) |  |
| CHS supplemental | Visit 5 | 1996-1997 | 11 (6-17) | 11 (6-17) | 10 (5-17) | 10 (5-17) | 10 (5-17) | 11 (6-18) | 12 (7-18) | 12 (7-18) | 12 (7-18) |  |
| CRIC | Visit 3 | 2013- 2015 | 7 (5 - 13) | 7 (5 - 14) | 7 (5 - 14) | 6 (4 - 13) | 6 (3 - 11) | 8 (5 - 14) | 8 (5 - 14) | 8 (5 - 14) | 7 (5 - 13) |  |
| DHS | DHS 1 Phase 1 | 2003-2007 | 15 (14 - 15) | 20 (19 - 20) | 20 (19 - 20) | 15 (14 - 15) | 15 (14 - 15) | 20 (20 - 20) | 20 (20 - 20) | 20 (19 - 20) | 15 (14 - 15) |  |
| ELSA-Brasil | Exam 1 | 2008 and 2010 | 4 (3-4) | 4 (3-4) | 4 (3-4) | 4 (3-4) | 8 (7-8) | 8 (7-8) | 8 (7-8) | 8 (7-8) | 8 (7-8) |  |
| FHS, original | Exam 7 | 1968-1971 | 24 (14 - 34) | 21 (11 - 31) | 20 (10 - 30) | 24 (14 - 34) | 23 (14 - 33) | 24 (15 - 34) | 24 (15 - 34) | 24 (15 - 34) | 22 (12 - 32) |  |
| FHS, offspring | Exam 1 | 1971-1975 | 42 (32 - 45) | 42 (28 - 45) | 41 (28 - 45) | 42 (32 - 45) | 40 (26 - 44) | 44 (34 - 46) | 44 (34 - 46) | 44 (34 - 46) | 42 (28 - 45) |  |
| FHS, 3^rd^ Generation | Exam 1 | 2002-2005 | 15 (13 - 16) | 15 (14 - 16) | 15 (14 - 16) | 15 (13 - 16) | 14 (13 - 15) | 15 (14 - 16) | 15 (14 - 16) | 15 (14 - 16) | 15 (14 - 16) |  |
| Health ABC | Year 1 | 1997-1998 | 13 (8 - 13) | 13 (8 - 13) | 12 (7 - 13) | NA | NA | 13 (8 - 13) | 13 (8 - 13) | 13 (8 - 13) | 13 (8 - 13) |  |
| JHS | Visit 1 | 2000-2004 | 14 (12 - 15) | 14 (13 - 15) | 14 (11 - 15) | 12 (11 - 12) | NA | 17 (16 - 18) | NA | 17 (16 - 18) | 14 (13 - 15) |  |
| MESA | Visit 1 | 2000-2002 | 17 (12 - 17) | 17 (14 - 18) | 17 (13 - 18) | 17 (12 - 17) | 14 (10 - 15) | 17 (15 - 18) | 17 (15 - 18) | 17 (15 - 18) | 17 (12 - 17) |  |
| MRFIT | Visit 2 | 1975-1976 | 6 (6 - 6) | 11 (10 - 11) | 11 (10 - 11) | NA | NA | 11 (10 - 11) | 11 (10 - 11) | 11 (10 - 11) | 6 (6 - 6) |  |
| MROS | Baseline | 2000 and 2002 | 10 (7-10) | 10 (6-10) | 10 (7-10) | 10 (7-10) | 13 (7-18) | 13 (8-19) | 13 (8-19) | 13 (8-19) | 13 (8-19) |  |
| REGARDS | Visit 1 | 2003-2007 | 8 (4 - 13) | 13 (7 - 19) | 12 (7 - 19) | 8 (4 - 12) | 13 (12 - 22) | 13 (7 - 20) | 13 (7 - 20) | 13 (7 - 20) | 8 (4 - 12) |  |
| RBS | Visit 4 | 1984 and 1987 | 12 (6 - 15) | 11 (6 - 14) | 11 (6 - 15) | 12 (7 - 15) | 9 (8 - 10) | 11 (7 - 14) | 11 (7 - 14) | 11 (7 - 14) | 11 (6 - 14) |  |
| SHS | Phase 1 | 1989-1991 | 30 (29 - 31) | 30 (26 - 31) | 29 (17 - 30) | 30 (29 - 31) | NA | 20 (10 - 29) | 20 (10 - 29) | 20 (10 - 29) | 30 (29 - 31) |  |
| SOF | Visit 1 | 1986 and 1988 | NA | NA | NA | NA | 18 (12-21) | 19 (13-21) | 19 (13-21) | 18 (12-21) | 15 (10-20) |  |
| SWAN | Visit 0 | 1996 and 1997 | 19 (11-19) | 19 (11-19) | 19 (13-19) | NA | 19 (11-19) | 19 (11-19) | 19 (13-19) | 19 (13-19) | 19 (13-19) |  |
| WHI Observational cohort | Baseline assessment | 1993-1998 | 17 (8-22) | 16 (8-22) | 17 (8-23) | NA | 21 (15-23) | 21 (16-23) | 22 (17-23) | 22 (17-23) | 22 (17-23) |  |
| WHI  clinical trial | Baseline assessment | 1993-1998 | 19 (9-23) | 18 (9-23) | 20 (10-23) | 5 (5-6) | 22 (16-23) | 22 (17-23) | 22 (18-23) | 22 (18-23) | 22 (18-23) |  |
| Total – CCC dataset | NA | 1958-2015 | 14.4 (8.1 - 23.0) | 14.8 (8.0 - 22.9) | 15.6 (9.0 - 23.2) | 8.7 (6.0 - 14.3) | 18.6 (11.2 - 23.4) | 19.3 (11.7 - 23.5) | 19.7 (12.5 - 23.6) | 19.9 (12.4 - 23.7) | 19.4 (12.3 - 23.5) |  |
| NA; Not available  Atherosclerosis Risk in Communities (ARIC) Study, 2) Baltimore Longitudinal Study of Aging (BLSA), 3) Coronary Artery Risk Development in Young Adults (CARDIA) Study, 4) Chronic Renal Insufficiency Cohort (CRIC) 5) Dallas Heart Study (DHS) 6-8) Framingham Heart Study (FHS), original, offspring and third generation 9) the Health, Aging and Body Composition Study (Health ABC) 10) Jackson Heart Study (JHS) 11) Multi-Ethnic Study of Atherosclerosis (MESA), 12) Multiple Risk Factor Intervention Trial (MRFIT) 13) the Reasons for Geographic and Racial Differences in Stroke Study (REGARDS) 14) Rancho Bernardo Study (RBS) 15) the Strong Heart Study (SHS)  MI: myocardial infarction; CHD: coronary heart disease; CVD: cardiovascular disease  ARIC: Atherosclerosis Risk in Communities Study, CARDIA: Coronary Artery Risk Development in Young Adults, CHS: Cardiovascular Health Study, DHS: Dallas Heart Study, FHSL: Framingham Heart Study, JHS: Jackson Heart Study, MESA: Multi-Ethnic Study of Atherosclerosis, MRFIT: The Multiple Risk Factor Intervention Trial, REGARDS: Reasons for Geographic and Racial Differences in Stroke, SHS: Strong Heart Study, BLSA: Baltimore Longitudinal Study of Aging, CRIC: Chronic Renal Insufficiency Cohort Study, ELSA-Brasil: Brazilian Longitudinal Study of Adult Health, Health ABC: Health Aging and Body Composition Study, MrOS: The Osteoporotic Fractures in Men Study, RBS: Rancho Bernardo Study of Healthy Aging, SOF: The Study of Osteoporotic Fractures, SWAN: Study of Women's Health Across the Nation, WHI: Women's Health Initiative. | | | | | | | | | | | | |
