# Supplementary material for: Association between cigarette smoking status, intensity, and cessation duration with long-term incidence of nine cardiovascular and mortality outcomes: The Cross-Cohort Collaboration (CCC)
Source: PLoS Med. 2025 Nov 18;22(11):e1004561. doi: 10.1371/journal.pmed.1004561 (PMC12626310; doi:10.1371/journal.pmed.1004561)
Supplement: S5 Table — (DOCX) [file pmed.1004561.s005.docx]

| **S5 Table. Association between smoking status and incidence of cardiovascular and mortality outcomes excluding patients reported baseline cardiovascular disease** | | | | |
| --- | --- | --- | --- | --- |
| **Outcome** | **Model** | **Never** | **Former** | **Current** |
| **MI** | Model 1 HR (95% CI) | 1.00 | 1.17 (1.13, 1.22) | 1.78 (1.70, 1.85) |
|  | Model 2 HR (95% CI) | 1.00 | 1.18 (1.14, 1.22) | 1.95 (1.86, 2.04) |
| **Stroke** | Model 1 HR (95% CI) | 1.00 | 1.12 (1.08, 1.16) | 1.55 (1.47, 1.62) |
|  | Model 2 HR (95% CI) | 1.00 | 1.12 (1.09, 1.16) | 1.71 (1.63, 1.80) |
| **CHD** | Model 1 HR (95% CI) | 1.00 | 1.16 (1.13, 1.19) | 1.82 (1.76, 1.89) |
|  | Model 2 HR (95% CI) | 1.00 | 1.16 (1.13, 1.19) | 2.02 (1.95, 2.10) |
| **CVD** | Model 1 HR (95% CI) | 1.00 | 1.13 (1.11, 1.15) | 1.76 (1.72, 1.81) |
|  | Model 2 HR (95% CI) | 1.00 | 1.13 (1.11, 1.15) | 1.97 (1.91, 2.02) |
| **Heart failure** | Model 1 HR (95% CI) | 1.00 | 1.22 (1.18, 1.26) | 1.68 (1.60, 1.75) |
|  | Model 2 HR (95% CI) | 1.00 | 1.22 (1.17, 1.26) | 2.00 (1.91, 2.09) |
| **Atrial fibrillation** | Model 1 HR (95% CI) | 1.00 | 1.12 (1.07, 1.17) | 1.39 (1.32, 1.47) |
|  | Model 2 HR (95% CI) | 1.00 | 1.10 (1.05, 1.15) | 1.55 (1.47, 1.64) |
| **Mortality Outcome** |  |  |  |  |
| **CHD mortality** | Model 1 HR (95% CI) | 1.00 | 1.17 (1.13, 1.21) | 2.00 (1.90, 2.10) |
|  | Model 2 HR (95% CI) | 1.00 | 1.16 (1.12, 1.21) | 2.30 (2.19, 2.42) |
| **CVD mortality** | Model 1 HR (95% CI) | 1.00 | 1.13 (1.10, 1.16) | 1.94 (1.87, 2.01) |
|  | Model 2 HR (95% CI) | 1.00 | 1.13 (1.11, 1.16) | 2.21 (2.13, 2.29) |
| **All-cause mortality** | Model 1 HR (95% CI) | 1.00 | 1.21 (1.19, 1.22) | 2.21 (2.17, 2.25) |
|  | Model 2 HR (95% CI) | 1.00 | 1.22 (1.20, 1.23) | 2.38 (2.34, 2.43) |
| Model 1 adjusted for age, sex, race and ethnicity, and education status.  Model 2 adjusted for age, sex, race and ethnicity, education status, body mass index, diabetes, hyperlipidemia, antihypertensive and lipid-lowering medication use, systolic blood pressure, diastolic blood pressure, and alcohol use.  Models include a shared frailty component for 'cohort' to account for intra-group correlation within the 22 unique cohorts  HR: Hazard ratio; CI: Confidence interval; MI: myocardial infarction; CHD: coronary heart disease; CVD: cardiovascular disease | | | | |
