# Supplementary material for: Association between cigarette smoking status, intensity, and cessation duration with long-term incidence of nine cardiovascular and mortality outcomes: The Cross-Cohort Collaboration (CCC)
Source: PLoS Med. 2025 Nov 18;22(11):e1004561. doi: 10.1371/journal.pmed.1004561 (PMC12626310; doi:10.1371/journal.pmed.1004561)
Supplement: S6 Table — (DOCX) [file pmed.1004561.s006.docx]

| **S6 Table. Association between smoking Status and Incidence of cardiovascular and Mortality Outcomes in participants older than 60 years old** | | | | |
| --- | --- | --- | --- | --- |
| **Outcome** | **Model** | **Never** | **Former** | **Current** |
| **MI** | Model 1 HR (95% CI) | 1.00 | 1.19 (1.14, 1.24) | 1.63 (1.53, 1.75) |
|  | Model 2 HR (95% CI) | 1.00 | 1.20 (1.15, 1.25) | 1.80 (1.68, 1.92) |
| **Stroke** | Model 1 HR (95% CI) | 1.00 | 1.11 (1.06, 1.15) | 1.36 (1.27, 1.45) |
|  | Model 2 HR (95% CI) | 1.00 | 1.11 (1.07, 1.15) | 1.49 (1.39, 1.59) |
| **CHD** | Model 1 HR (95% CI) | 1.00 | 1.18 (1.14, 1.21) | 1.72 (1.64, 1.81) |
|  | Model 2 HR (95% CI) | 1.00 | 1.18 (1.14, 1.22) | 1.92 (1.83, 2.02) |
| **CVD** | Model 1 HR (95% CI) | 1.00 | 1.14 (1.12, 1.17) | 1.66 (1.60, 1.72) |
|  | Model 2 HR (95% CI) | 1.00 | 1.15 (1.12, 1.17) | 1.84 (1.77, 1.91) |
| **Heart failure** | Model 1 HR (95% CI) | 1.00 | 1.23 (1.17, 1.28) | 1.60 (1.50, 1.70) |
|  | Model 2 HR (95% CI) | 1.00 | 1.23 (1.18, 1.29) | 1.90 (1.78, 2.04) |
| **Atrial fibrillation** | Model 1 HR (95% CI) | 1.00 | 1.11 (1.05, 1.18) | 1.30 (1.19, 1.42) |
|  | Model 2 HR (95% CI) | 1.00 | 1.10 (1.04, 1.16) | 1.42 (1.30, 1.55) |
| **Mortality Outcome** |  |  |  |  |
| **CHD mortality** | Model 1 HR (95% CI) | 1.00 | 1.17 (1.13, 1.21) | 1.90 (1.79, 2.03) |
|  | Model 2 HR (95% CI) | 1.00 | 1.17 (1.13, 1.22) | 2.19 (2.06, 2.34) |
| **CVD mortality** | Model 1 HR (95% CI) | 1.00 | 1.14 (1.11, 1.17) | 1.86 (1.78, 1.95) |
|  | Model 2 HR (95% CI) | 1.00 | 1.15 (1.12, 1.18) | 2.11 (2.01, 2.21) |
| **All-cause mortality** | Model 1 HR (95% CI) | 1.00 | 1.22 (1.20, 1.24) | 2.17 (2.12, 2.22) |
|  | Model 2 HR (95% CI) | 1.00 | 1.23 (1.22, 1.25) | 2.34 (2.28, 2.39) |
| Model 1 adjusted for age, sex, race and ethnicity, and education status.  Model 2 adjusted for age, sex, race and ethnicity, education status, body mass index, diabetes, hyperlipidemia, antihypertensive and lipid-lowering medication use, systolic blood pressure, diastolic blood pressure, history of coronary heart disease at baseline, and alcohol use.  Models include a shared frailty component for 'cohort' to account for intra-group correlation within the 22 unique cohorts  HR: Hazard ratio; CI: Confidence interval; MI: myocardial infarction; CHD: coronary heart disease; CVD: cardiovascular disease | | | | |
