# Supplementary material for: Association between cigarette smoking status, intensity, and cessation duration with long-term incidence of nine cardiovascular and mortality outcomes: The Cross-Cohort Collaboration (CCC)
Source: PLoS Med. 2025 Nov 18;22(11):e1004561. doi: 10.1371/journal.pmed.1004561 (PMC12626310; doi:10.1371/journal.pmed.1004561)
Supplement: S8 Table — (DOCX) [file pmed.1004561.s008.docx]

| **S8 Table.**  **Association between pack years with cardiovascular outcomes among former cigarette users** | | | | | | |
| --- | --- | --- | --- | --- | --- | --- |
|  | **Per 10 increments of**  **Pack-years ^a^** | **Pack-years categories** | | | | |
|  |  | **Never Smokers**  **[ Reference] ^b^** | **≤ 5 pack-years**  **N= 28951** | **6 - 10 pack years**  **N= 12618** | **11 - 20 pack-years**  **N= 19824** | **> 20 pack-years**  **N= 38521** |
| **CV outcomes** | |  | | | | |
| **MI** |  |  |  |  |  |  |
| Model 1 HR (95% CI) | **1.032 (1.023, 1.041)** | **1** | 0.92 (0.86, 0.98) | 0.97 (0.89, 1.06) | **1.10 (1.02, 1.17)** | **1.38 (1.31, 1.44)** |
| Model 2 HR (95% CI) | **1.027 (1.017, 1.038)** | **1** | 0.95 (0.88, 1.02) | 1.02 (0.93, 1.12) | **1.14 (1.06, 1.22)** | **1.33 (1.26, 1.40)** |
| **Stroke** |  |  |  |  |  |  |
| Model 1 HR (95% CI) | **1.032 (1.023, 1.042)** | **1** | 0.96 (0.91, 1.02) | 1.00 (0.92, 1.08) | **1.13 (1.06, 1.20)** | **1.28 (1.22, 1.34)** |
| Model 2 HR (95% CI) | **1.023 (****1.012, 1.034)** | **1** | 0.99 (0.93, 1.06) | 1.04 (0.95, 1.13) | **1.18 (1.10, 1.26)** | **1.23 (1.17, 1.29)** |
| **CHD** |  |  |  |  |  |  |
| Model 1 HR (95% CI) | **1.043 (1.037, 1.050)** | **1** | 0.92 (0.88, 0.97) | 0.97 (0.91, 1.03) | **1.09 (1.04, 1.15)** | **1.36 (1.31, 1.41)** |
| Model 2 HR (95% CI) | **1.035 (1.028, 1.043)** | **1** | 0.97 (0.92, 1.02) | 1.01 (0.94, 1.08) | **1.15 (1.09, 1.21)** | **1.31 (1.26, 1.36)** |
| **CVD** |  |  |  |  |  |  |
| Model 1 HR (95% CI) | **1.039 (1.034, 1.044)** | **1** | 0.94 (0.90, 0.97) | 1.00 (0.95, 1.05) | **1.08 (1.04, 1.12)** | **1.31 (1.27, 1.34)** |
| Model 2 HR (95% CI) | **1.030 (1.024, 1.036)** | **1** | 0.97 (0.94, 1.01) | 1.04 (0.99, 1.10) | **1.13 (1.09, 1.18)** | **1.27 (1.24, 1.31)** |
| **Heart failure** |  |  |  |  |  |  |
| Model 1 HR (95% CI) | **1.044 (1.036, 1.051)** | **1** | 0.88 (0.81, 0.95) | 0.92 (0.83, 1.02) | **1.08 (1.00, 1.16)** | **1.48 (1.41, 1.56)** |
| Model 2 HR (95% CI) | **1.041 (1.032, 1.051)** | **1** | 0.91 (0.83, 0.99) | 1.01 (0.90, 1.12) | **1.16 (1.07, 1.26)** | **1.43 (1.35, 1.51)** |
| **AFib** |  |  |  |  |  |  |
| Model 1 HR (95% CI) | **1.042 (1.025, 1.060)** | **1** | 0.99 (0.89, 1.10) | **1.14 (1.02, 1.29)** | 1.04 (0.94, 1.14) | **1.23 (1.15, 1.32)** |
| Model 2 HR (95% CI) | **1.035 (1.016, 1.055)** | **1** | 1.01 (0.90, 1.13) | **1.17 (1.03, 1.33)** | 1.08 (0.97, 1.20) | **1.19 (1.10, 1.28)** |
| **Mortality Outcomes** | | | | | | |
| **CHD Mortality** |  |  |  |  |  |  |
| Model 1 HR (95% CI) | **1.057 (1.049, 1.065)** | **1** | 0.92 (0.86, 0.97) | 0.94 (0.87, 1.02) | **1.10 (1.03, 1.17)** | **1.40 (1.34, 1.46)** |
| Model 2 HR (95% CI) | **1.047 (1.037, 1.057)** | **1** | 0.97 (0.91, 1.04) | 1.00 (0.91, 1.10) | **1.16 (1.09, 1.25)** | **1.33 (1.27, 1.40)** |
| **CVD Mortality** |  |  |  |  |  |  |
| Model 1 HR (95% CI) | **1.049 (1.043, 1.055)** | **1** | 0.92 (0.88, 0.96) | 0.99 (0.93, 1.05) | **1.06 (1.01, 1.11)** | **1.35 (1.31, 1.39)** |
| Model 2 HR (95% CI) | **1.040 (1.032, 1.047)** | **1** | 0.96 (0.92, 1.01) | 1.03 (0.97, 1.11) | **1.12 (1.06, 1.18)** | **1.31 (1.26, 1.36)** |
| **All-cause mortality** |  |  |  |  |  |  |
| Model 1 HR (95% CI) | **1.062 (1.060, 1.065)** | **1** | 0.95 (0.93, 0.98) | 1.02 (0.99, 1.05) | **1.11 (1.09, 1.14)** | **1.50 (1.47, 1.52)** |
| Model 2 HR (95% CI) | **1.062 (1.058, 1.065)** | **1** | 0.98 (0.96, 1.00) | **1.04 (****1.01, 1.08)** | **1.14 (1.12, 1.17)** | **1.49 (1.46, 1.51)** |
| Model 1 adjusted for age, sex, race and ethnicity, and education status.  Model 2 adjusted for age, sex, race and ethnicity, education status, body mass index, diabetes, hyperlipidemia, antihypertensive and lipid-lowering medication use, systolic blood pressure, diastolic blood pressure, history of coronary heart disease at baseline, and alcohol use.  Models include a shared frailty component for 'cohort' to account for intra-group correlation within the 22 unique cohorts  ^a^ Pack-years was considered as a continuous variable.  **^b^** This is the reference group (i.e., never-smokers) for the categorical analysis.  HR: Hazard ratio; CI: Confidence interval; MI: myocardial infarction; AFib: Atrial fibrillation; CHD: coronary heart disease; CVD: cardiovascular disease | | | | | | |
