# Supplementary material for: Association between cigarette smoking status, intensity, and cessation duration with long-term incidence of nine cardiovascular and mortality outcomes: The Cross-Cohort Collaboration (CCC)
Source: PLoS Med. 2025 Nov 18;22(11):e1004561. doi: 10.1371/journal.pmed.1004561 (PMC12626310; doi:10.1371/journal.pmed.1004561)
Supplement: S9 Table — (DOCX) [file pmed.1004561.s009.docx]

| **S9 Table.**  **Association between pack years with cardiovascular outcomes among current cigarette users** | | | | | | |
| --- | --- | --- | --- | --- | --- | --- |
|  | **Per 10 increments of**  **Pack years ^a^** | **Pack year categories** | | | | |
|  |  | **Never Smokers**  **[ Reference] ^b^** | **≤ 5 pack year**  **N= 4862** | **6 - 10 pack year**  **N = 3607** | **11 - 20 pack year**  **N= 6947** | **> 20 pack year**  **N= 27642** |
| **CV outcomes** | |  | | | | |
| **MI** |  |  |  |  |  |  |
| Model 1 HR (95% CI) | **1.028 (1.017, 1.038)** | **1** | **1.25 (1.11, 1.41)** | **1.60 (1.42, 1.80)** | **1.57 (1.44, 1.72)** | **1.82 (1.73, 1.90)** |
| Model 2 HR (95% CI) | **1.030 (1.019, 1.042)** | **1** | **1.36 (1.20, 1.55)** | **1.66 (1.45, 1.89)** | **1.70 (1.55, 1.86)** | **2.00 (1.90, 2.10)** |
| **Stroke** |  |  |  |  |  |  |
| Model 1 HR (95% CI) | **1.017 (1.003, 1.030)** | **1** | **1.16 (1.01, 1.33)** | **1.34 (1.17, 1.54)** | **1.57 (1.43, 1.73)** | **1.58 (1.50, 1.67)** |
| Model 2 HR (95% CI) | **1.024 (1.010, 1.039)** | **1** | **1.28 (1.11, 1.48)** | **1.41 (1.21, 1.64)** | **1.71 (1.54, 1.89)** | **1.78 (1.68, 1.89)** |
| **CHD** |  |  |  |  |  |  |
| Model 1 HR (95% CI) | **1.027 (1.018, 1.036)** | **1** | **1.27 (1.15, 1.41)** | **1.64 (1.49, 1.81)** | **1.66 (1.55, 1.78)** | **1.88 (1.81, 1.96)** |
| Model 2 HR (95% CI) | **1.027 (1.018, 1.037)** | **1** | **1.41 (1.26, 1.57)** | **1.71 (1.53, 1.90)** | **1.86 (1.72, 2.01)** | **2.11 (2.02, 2.20)** |
| **CVD** |  |  |  |  |  |  |
| Model 1 HR (95% CI) | **1.021 (1.014, 1.028)** | **1** | **1.26 (1.17, 1.37)** | **1.57 (1.46, 1.70)** | **1.66 (1.57, 1.76)** | **1.83 (1.78, 1.89)** |
| Model 2 HR (95% CI) | **1.024 (****1.016, 1.032)** | **1** | **1.39 (1.28, 1.52)** | **1.66 (1.53, 1.81)** | **1.85 (1.74, 1.96)** | **2.06 (1.99, 2.13)** |
| **Heart failure** |  |  |  |  |  |  |
| Model 1 HR (95% CI) | **1.027 (1.016, 1.038)** | **1** | **1.31 (1.16, 1.48)** | **1.43 (1.27, 1.61)** | **1.40 (1.28, 1.53)** | **1.74 (1.65, 1.82)** |
| Model 2 HR (95% CI) | **1.027 (1.016, 1.039)** | **1** | **1.60 (1.41, 1.82)** | **1.68 (1.47, 1.92)** | **1.66 (1.50, 1.83)** | **2.12 (2.01, 2.24)** |
| **AFib** |  |  |  |  |  |  |
| Model 1 HR (95% CI) | **1.043 (1.024, 1.062)** | **1** | 0.95 (0.82, 1.11) | **1.12 (0.96, 1.31)** | **1.28 (1.15, 1.43)** | **1.45 (1.37, 1.54)** |
| Model 2 HR (95% CI) | **1.041 (****1.023, 1.059)** | **1** | 1.09 (0.93, 1.27) | **1.22 (1.04, 1.43)** | **1.46 (1.31, 1.63)** | **1.64 (1.54, 1.74)** |
| **Mortality Outcomes** | | | | | | |
| **CHD Mortality** |  |  |  |  |  |  |
| Model 1 HR (95% CI) | **1.030 (1.018, 1.042)** | **1** | **1.28 (1.11, 1.49)** | **1.78 (1.55, 2.03)** | **1.75 (1.58, 1.94)** | **2.12 (2.01, 2.24)** |
| Model 2 HR (95% CI) | **1.036 (1.022, 1.050)** | **1** | **1.46 (1.24, 1.72)** | **1.86 (1.59, 2.18)** | **2.08 (1.86, 2.33)** | **2.45 (2.31, 2.60)** |
| **CVD Mortality** |  |  |  |  |  |  |
| Model 1 HR (95% CI) | **1.025 (1.016, 1.034)** | **1** | **1.25 (1.12, 1.40)** | **1.68 (1.52, 1.87)** | **1.73 (1.60, 1.87)** | **2.08 (2.00, 2.17)** |
| Model 2 HR (95% CI) | **1.031 (1.021, 1.041)** | **1** | **1.44 (1.28, 1.62)** | **1.81 (1.61, 2.03)** | **2.01 (1.84, 2.19)** | **2.37 (2.27, 2.48)** |
| **All-cause mortality** |  |  |  |  |  |  |
| Model 1 HR (95% CI) | **1.041 (1.036, 1.045)** | **1** | **1.33 (1.25, 1.40)** | **1.68 (1.59, 1.77)** | **1.81 (1.73, 1.88)** | **2.47 (2.42, 2.52)** |
| Model 2 HR (95% CI) | **1.046 (1.041, 1.050)** | **1** | **1.40 (1.31, 1.49)** | **1.77 (1.67, 1.89)** | **1.99 (1.90, 2.08)** | **2.71 (2.65, 2.77)** |
| Model 1 adjusted for age, sex, race and ethnicity, and education status.  Model 2 adjusted for age, sex, race and ethnicity, education status, body mass index, diabetes, hyperlipidemia, antihypertensive and lipid-lowering medication use, systolic blood pressure, diastolic blood pressure, history of coronary heart disease at baseline, and alcohol use.  Models include a shared frailty component for 'cohort' to account for intra-group correlation within the 22 unique cohorts  ^a^ Pack-years was considered as a continuous variable.  **^b^** This is the reference group (i.e., never-smokers) for the categorical analysis.  HR: Hazard ratio; CI: Confidence interval; MI: myocardial infarction; AFib: Atrial fibrillation; CHD: coronary heart disease; CVD: cardiovascular disease | | | | | | |
