# Supplementary material for: Association between cigarette smoking status, intensity, and cessation duration with long-term incidence of nine cardiovascular and mortality outcomes: The Cross-Cohort Collaboration (CCC)
Source: PLoS Med. 2025 Nov 18;22(11):e1004561. doi: 10.1371/journal.pmed.1004561 (PMC12626310; doi:10.1371/journal.pmed.1004561)
Supplement: S10 Table — (DOCX) [file pmed.1004561.s010.docx]

| **S10 Table. Association between the number of cigarettes per day with cardiovascular outcomes among current cigarette users** | | | | | | | | | | |
| --- | --- | --- | --- | --- | --- | --- | --- | --- | --- | --- |
| **Outcome** | **Model** | **Never** | **≤1 CPD** | **2-5 CPD** | **6-10 CPD** | **11-15 CPD** | **16-20 CPD** | **>20-25 CPD** | **>25-30 CPD** | **>30 CPD** |
| **MI** | Model 1 HR (95% CI) | 1.00 (ref) | 1.23 (0.99 - 1.52) | 1.43 (1.28 - 1.60) | 1.67 (1.54 - 1.82) | 1.53 (1.34 - 1.75) | 1.91 (1.79 - 2.03) | 2.16 (1.78 - 2.61) | 2.05 (1.86 - 2.27) | 1.99 (1.82 - 2.18) |
|  | Model 2 HR (95% CI) | 1.00 (ref) | 1.42 (1.13 - 1.77) | 1.50 (1.32 - 1.70) | 1.91 (1.75 - 2.08) | 1.74 (1.51 - 2.02) | 2.07 (1.94 - 2.21) | 2.33 (1.93 - 2.83) | 2.23 (2.02 - 2.47) | 2.13 (1.94 - 2.34) |
| **Stroke** | Model 1 HR (95% CI) | 1.00 (ref) | 1.18 (0.94 - 1.49) | 1.28 (1.13 - 1.45) | 1.52 (1.40 - 1.66) | 1.40 (1.19 - 1.64) | 1.64 (1.53 - 1.76) | 1.57 (1.18 - 2.10) | 1.66 (1.46 - 1.89) | 1.62 (1.43 - 1.84) |
|  | Model 2 HR (95% CI) | 1.00 (ref) | 1.26 (0.98 - 1.63) | 1.41 (1.23 - 1.61) | 1.67 (1.52 - 1.83) | 1.69 (1.42 - 1.99) | 1.83 (1.70 - 1.97) | 1.56 (1.16 - 2.10) | 1.80 (1.57 - 2.06) | 1.77 (1.55 - 2.02) |
| **CHD** | Model 1 HR (95% CI) | 1.00 (ref) | 1.24 (1.04 - 1.47) | 1.41 (1.29 - 1.55) | 1.68 (1.57 - 1.79) | 1.65 (1.48 - 1.85) | 1.99 (1.89 - 2.09) | 2.11 (1.78 - 2.50) | 2.17 (1.99 - 2.36) | 2.07 (1.91 - 2.25) |
|  | Model 2 HR (95% CI) | 1.00 (ref) | 1.48 (1.24 - 1.78) | 1.51 (1.36 - 1.67) | 1.94 (1.81 - 2.08) | 1.91 (1.69 - 2.16) | 2.18 (2.07 - 2.30) | 2.31 (1.94 - 2.74) | 2.38 (2.19 - 2.60) | 2.23 (2.06 - 2.42) |
| **CVD** | Model 1 HR (95% CI) | 1.00 (ref) | 1.26 (1.10 - 1.44) | 1.39 (1.29 - 1.49) | 1.61 (1.54 - 1.70) | 1.62 (1.48 - 1.76) | 1.93 (1.85 - 2.00) | 1.96 (1.70 - 2.26) | 2.02 (1.88 - 2.16) | 2.03 (1.90 - 2.17) |
|  | Model 2 HR (95% CI) | 1.00 (ref) | 1.47 (1.28 - 1.70) | 1.50 (1.38 - 1.62) | 1.83 (1.73 - 1.93) | 1.87 (1.69 - 2.06) | 2.12 (2.03 - 2.21) | 2.11 (1.83 - 2.44) | 2.22 (2.07 - 2.39) | 2.22 (2.07 - 2.37) |
| **HF** | Model 1 HR (95% CI) | 1.00 (ref) | 1.53 (1.25 - 1.86) | 1.33 (1.19 - 1.48) | 1.57 (1.44 - 1.70) | 1.59 (1.41 - 1.81) | 1.82 (1.71 - 1.94) | 1.80 (1.43 - 2.27) | 1.91 (1.70 - 2.14) | 1.93 (1.73 - 2.15) |
|  | Model 2 HR (95% CI) | 1.00 (ref) | 2.07 (1.69 - 2.54) | 1.57 (1.39 - 1.78) | 1.90 (1.74 - 2.08) | 1.98 (1.72 - 2.27) | 2.22 (2.08 - 2.38) | 2.09 (1.66 - 2.64) | 2.25 (1.99 - 2.53) | 2.14 (1.92 - 2.39) |
| **AFib** | Model 1 HR (95% CI) | 1.00 (ref) | 1.01 (0.77 - 1.32) | 1.12 (0.97 - 1.29) | 1.36 (1.22 - 1.52) | 1.37 (1.18 - 1.59) | 1.44 (1.32 - 1.56) | 1.32 (1.02 - 1.69) | 1.61 (1.42 - 1.84) | 1.75 (1.54 - 1.98) |
|  | Model 2 HR (95% CI) | 1.00 (ref) | 1.16 (0.87 - 1.53) | 1.26 (1.09 - 1.45) | 1.54 (1.37 - 1.72) | 1.64 (1.41 - 1.91) | 1.62 (1.49 - 1.77) | 1.47 (1.14 - 1.89) | 1.84 (1.61 - 2.10) | 1.89 (1.66 - 2.15) |
| **CHD Mortality** | Model 1 HR (95% CI) | 1.00 (ref) | 1.37 (1.09 - 1.73) | 1.43 (1.26 - 1.63) | 1.80 (1.65 - 1.96) | 1.93 (1.65 - 2.26) | 2.32 (2.16 - 2.49) | 1.81 (1.37 - 2.40) | 2.42 (2.14 - 2.73) | 2.39 (2.12 - 2.69) |
|  | Model 2 HR (95% CI) | 1.00 (ref) | 1.76 (1.38 - 2.24) | 1.55 (1.34 - 1.79) | 2.11 (1.92 - 2.32) | 2.38 (1.98 - 2.85) | 2.60 (2.41 - 2.82) | 2.07 (1.56 - 2.75) | 2.74 (2.41 - 3.11) | 2.70 (2.39 - 3.04) |
| **CVD Mortality** | Model 1 HR (95% CI) | 1.00 (ref) | 1.32 (1.11 - 1.57) | 1.43 (1.30 - 1.57) | 1.72 (1.61 - 1.84) | 1.80 (1.60 - 2.03) | 2.22 (2.11 - 2.34) | 1.89 (1.54 - 2.33) | 2.24 (2.04 - 2.47) | 2.30 (2.10 - 2.51) |
|  | Model 2 HR (95% CI) | 1.00 (ref) | 1.67 (1.39 - 2.00) | 1.57 (1.41 - 1.75) | 1.99 (1.85 - 2.14) | 2.06 (1.79 - 2.37) | 2.48 (2.34 - 2.63) | 2.48 (2.35 - 2.61) | 2.14 (1.74 - 2.64) | 2.52 (2.28 - 2.78) |
| **All-cause Mortality** | Model 1 HR (95% CI) | 1.00 (ref) | 1.36 (1.24 - 1.49) | 1.53 (1.45 - 1.60) | 1.95 (1.89 - 2.01) | 2.09 (1.96 - 2.22) | 2.52 (2.45 - 2.59) | 2.40 (2.15 - 2.68) | 3.00 (2.86 - 3.14) | 2.98 (2.84 - 3.12) |
|  | Model 2 HR (95% CI) | 1.00 (ref) | 1.51 (1.37 - 1.67) | 1.60 (1.52 - 1.69) | 2.14 (2.07 - 2.22) | 2.30 (2.14 - 2.47) | 2.75 (2.67 - 2.83) | 2.53 (2.26 - 2.83) | 3.21 (3.06 - 3.37) | 3.18 (3.03 - 3.33) |
| Model 1 adjusted for age, sex, race and ethnicity, and education status.  Model 2 adjusted for age, sex, race and ethnicity, education status, body mass index, diabetes, hyperlipidemia, antihypertensive and lipid-lowering medication use, systolic blood pressure, diastolic blood pressure, history of coronary heart disease at baseline, and alcohol use.  Models include a shared frailty component for 'cohort' to account for intra-group correlation within the 22 unique cohorts  **^a^** number of cigarettes per day was considered as a continuous variable.  the reference group for the categorical analysis is never smokers  HR: Hazard ratio; CI: Confidence interval; CPD: cigarettes per day, MI: myocardial infarction; AFib: Atrial fibrillation; CHD: coronary heart disease; CVD: cardiovascular disease | | | | | | | | | | |
