# Supplementary figures and images for: Association between cigarette smoking status, intensity, and cessation duration with long-term incidence of nine cardiovascular and mortality outcomes: The Cross-Cohort Collaboration (CCC)
Source: PLoS Med. 2025 Nov 18;22(11):e1004561. doi: 10.1371/journal.pmed.1004561 (PMC12626310; doi:10.1371/journal.pmed.1004561)

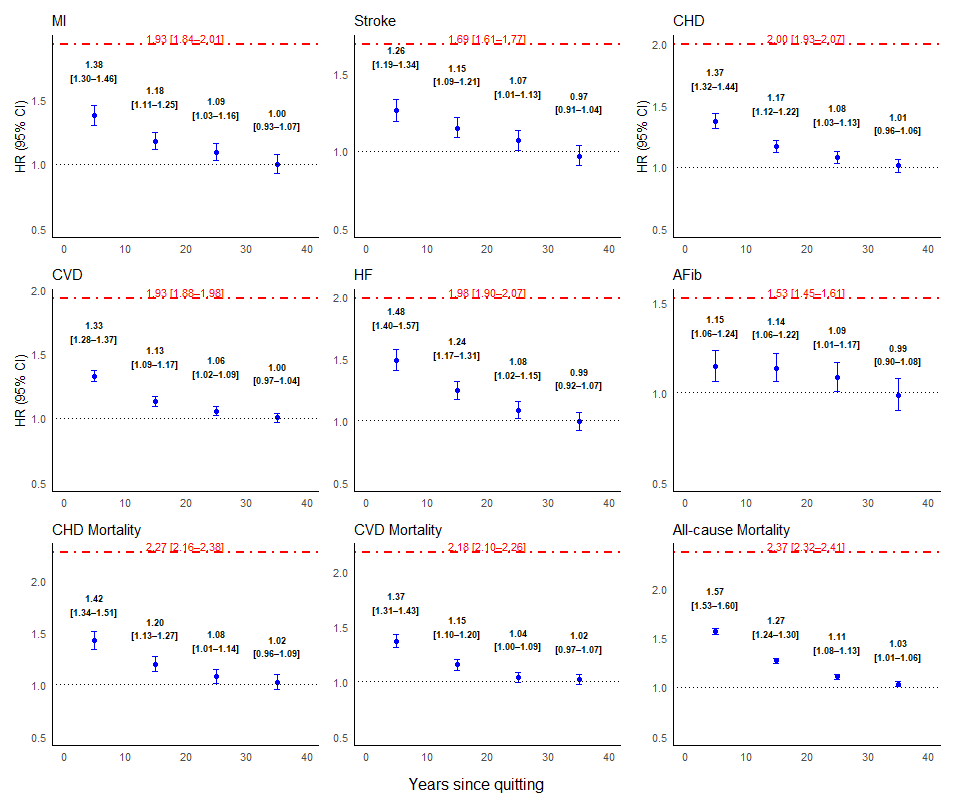

Supplement: S1 Fig — MI, myocardial infarction; CHD, coronary heart disease; CVD, cardiovascular disease; HF, heart failure, AFib, atrial fibrillation. Current smokers presented with a red dot-dashed line. The reference group is never-smokers presented with a dotted black line. (DOCX) [file pmed.1004561.s011.docx]

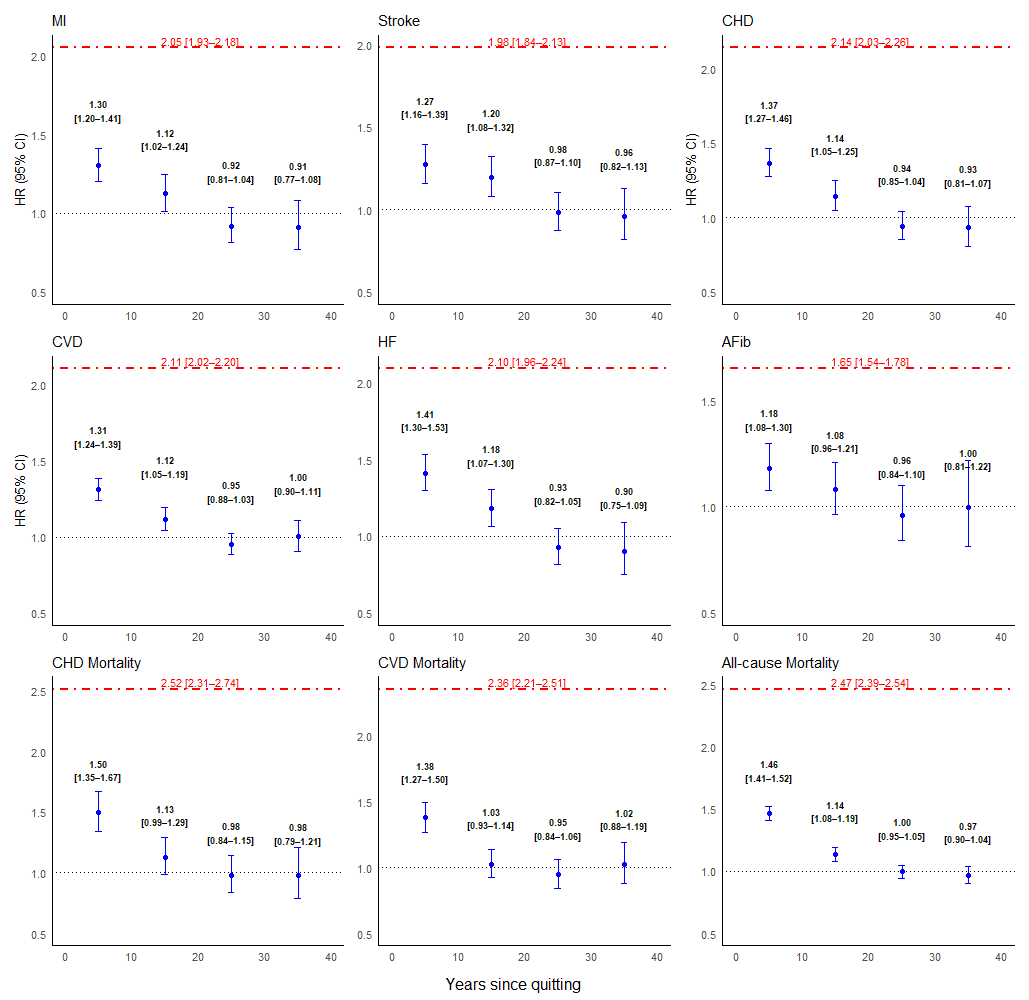

Supplement: S2 Fig — MI, myocardial infarction; CHD, coronary heart disease; CVD, cardiovascular disease; HF, heart failure, AFib, atrial fibrillation. Current smokers presented with a red dot-dashed line. The reference group is never-smokers presented with a dotted black line. (DOCX) [file pmed.1004561.s012.docx]

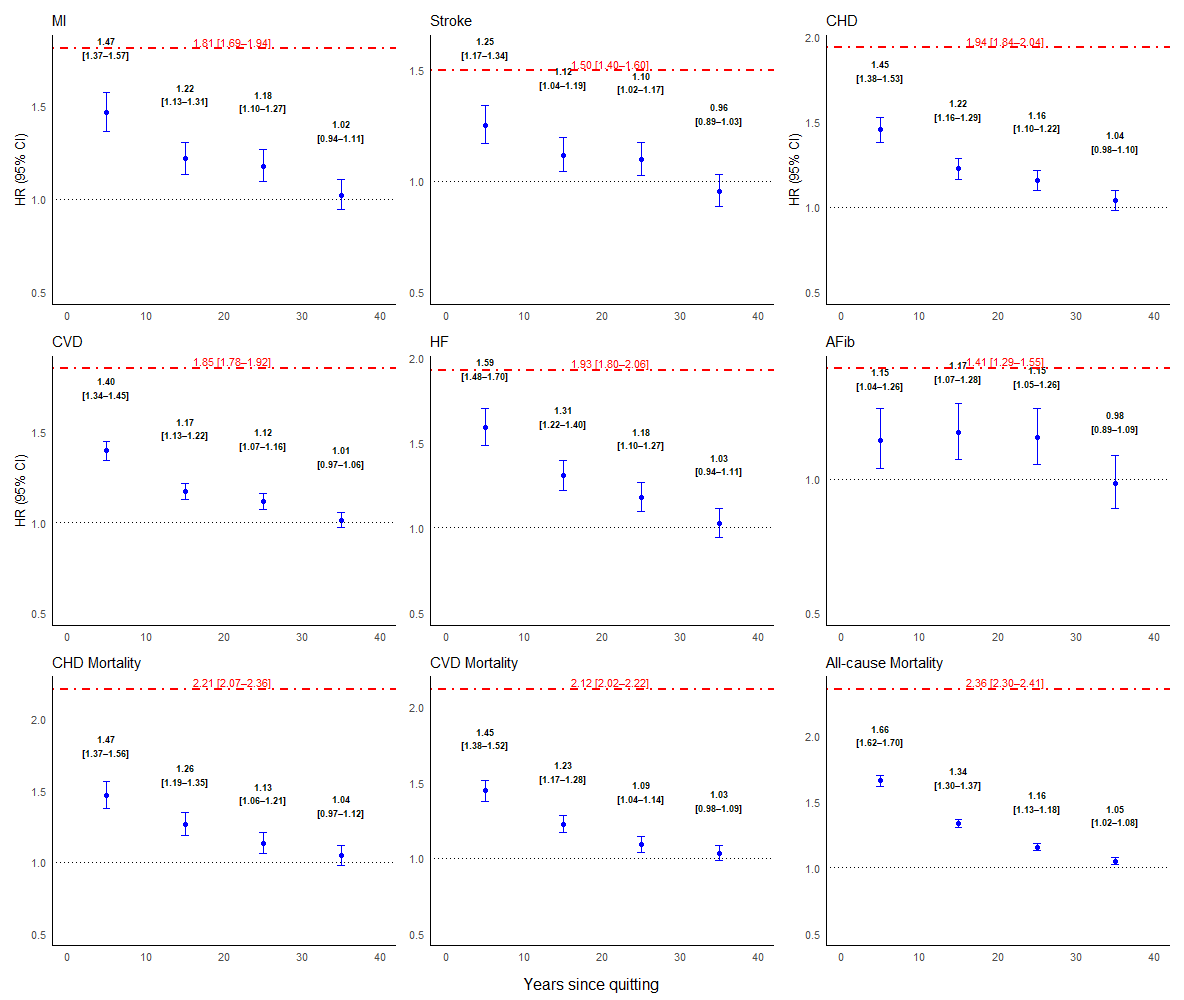

Supplement: S3 Fig — MI, myocardial infarction; CHD, coronary heart disease; CVD, cardiovascular disease; HF, heart failure, AFib, atrial fibrillation. Current smokers presented with a red dot-dashed line. The reference group is never-smokers presented with a dotted black line. (DOCX) [file pmed.1004561.s013.docx]
